# Inductive biases of neural network modularity in spatial navigation

**DOI:** 10.1126/sciadv.adk1256

**Published:** 2024-07-19

**Authors:** Ruiyi Zhang, Xaq Pitkow, Dora E. Angelaki

**Affiliations:** ^1^ Tandon School of Engineering, New York University, New York, NY, USA.; ^2^ Neuroscience Institute, Carnegie Mellon University, Pittsburgh, PA, USA.; ^3^ Department of Machine Learning, Carnegie Mellon University, Pittsburgh, PA, USA.; ^4^ Department of Neuroscience, Baylor College of Medicine, Houston, TX, USA.; ^5^ Department of Electrical and Computer Engineering, Rice University, Houston, TX, USA.; ^6^ Center for Neuroscience and Artificial Intelligence, Baylor College of Medicine, Houston, TX, USA.; ^7^ Center for Neural Science, New York University, New York, NY, USA.

## Abstract

The brain may have evolved a modular architecture for daily tasks, with circuits featuring functionally specialized modules that match the task structure. We hypothesize that this architecture enables better learning and generalization than architectures with less specialized modules. To test this, we trained reinforcement learning agents with various neural architectures on a naturalistic navigation task. We found that the modular agent, with an architecture that segregates computations of state representation, value, and action into specialized modules, achieved better learning and generalization. Its learned state representation combines prediction and observation, weighted by their relative uncertainty, akin to recursive Bayesian estimation. This agent’s behavior also resembles macaques’ behavior more closely. Our results shed light on the possible rationale for the brain’s modularity and suggest that artificial systems can use this insight from neuroscience to improve learning and generalization in natural tasks.

## Teaser

Modular neural architectures allow reinforcement learning agents to achieve better learning and generalization in navigation.

## INTRODUCTION

Accurate generalization beyond training tasks requires correct prior knowledge of the task structure (*1*). However, as Hume (*2*) famously highlighted in his “problem of induction,” one’s prior knowledge can be fallacious, leading to unsuccessful generalization. Nevertheless, animals have the ability to efficiently acquire the structure of their daily tasks as prior knowledge for unencountered tasks outside their typical domain (*1*, *3*–*5*). This remarkable ability may stem from the brain’s innate biases evolved for their daily tasks (*1*, *6*, *7*).

In theory, numerous solutions exist for a given task. For instance, one solution may involve comprehending the task’s underlying structure, i.e., understanding how data are generated from latent variables (*8*–*11*), whereas another could rely on memorizing all input-output pairs. Although both can produce positive results with sufficient training, a solution that understands the task structure is expected to exhibit greater data efficiency in mastering the training task and can generalize to unseen, structurally similar tasks; in contrast, rote memorization requires seeing all training examples and lacks generalizability. Every learning system for a task, whether biological or artificial, has a bias that favors some solutions over others, known as the inductive bias. For a neural network, its architecture defines a crucial aspect of this bias (*6*, *8*, *12*). To prioritize solutions that learn the task structure and support generalization, the inductive bias must be tailored to the specific tasks of interest, as there is no universal inductive bias suitable for all tasks [no-free-lunch (NFL) theorem] (*13*). Although we understand why inductive biases are important, it is still unclear how to tailor a useful one.

The remarkable ability of animals to rapidly learn and generalize in natural tasks suggests that their brains are indeed endowed with suitable inductive biases for these tasks. One perspective suggests that the brain may have evolved an inductive bias for a modular architecture featuring functionally specialized modules (*14*–*18*). Each module specializes in a specific aspect or a subset of task variables, collectively covering all demanding computations of the task. We hypothesize that this architecture enables higher efficiency in learning the structure of natural tasks and better generalization in tasks with a similar structure than those with less specialized modules.

Previous theoretical works (*8*–*11*) have outlined the potential rationale for this architecture: Data generated from natural tasks typically stem from the latent distribution of multiple task variables. Decomposing the task and learning these variables in distinct modules allow a better understanding of the relationships among these variables and therefore the data generation process. This modularization also promotes hierarchical computation, where independent variables are initially computed and then forwarded to other modules specialized in computing dependent variables. Hierarchical computation is a crucial factor contributing to the success of deep neural networks (*19*). Note that “modular” may take on different meanings in different contexts. Here, it specifically refers to architectures with multiple modules, each specializing in one or a subset of the desired task variables. Architectures with multiple modules lacking enforced specialization in computing variables do not meet the criteria for modular in our context.

To test our hypothesis, it is essential to select a natural task and compare a modular architecture designed for the task against alternative architectures. We chose a naturalistic virtual navigation task previously used to investigate the neural computations underlying macaques’ flexible behaviors (*20*, *21*), where subjects are required to steer toward a transiently visible target using optic flow cues. Subjects benefit from mentally computing multiple variables and understanding their dependencies, including an internal state representation of the outside world (a “belief”) given partial and noisy sensory cues, the motor commands (actions) used to control a joystick for navigation based on this belief, and the value of the action for the belief state (*22*).

We therefore designed a modular architecture tailored for this task, comprising dedicated modules that facilitate the recursive computation of beliefs using observations and predictions. It also includes specialized modules to compute actions and values based on the computed beliefs. This design not only promotes the modular computation of distinct variables but also establishes a hierarchical computation flow reflecting the dependencies among variables (e.g., action and value depend on belief). We also designed a set of alternative architectures for comparison, each featuring modules with weaker specializations for particular task variables.

To train artificial agents using these architectures, we used reinforcement learning (RL) (*23*) with sparse reward signals, similar to the training of macaques. We found that our modular architecture is better suited for mastering our task than alternative architectures. It demonstrated superior efficiency in learning a belief update rule, akin to recursive Bayesian estimation (*24*), compared to other architectures. The modular agent’s belief is updated by weighing the prior prediction from a motor efference copy against a likelihood derived from visual observation of states. The reliability of the two sources affects how these factors are combined, with the more reliable source assigned a higher weight. Furthermore, we will show that the learned control action of the modular agent reflects a more animal-like and efficient behavior than actions from alternative architectures.

After training, we proceeded to evaluate these agents in two previously unencountered tasks derived from the training task. One manipulated the sensorimotor mapping from joystick movements to subjects’ movements in the environment. The other randomly applied passive perturbations to subjects’ movements. Macaques demonstrated immediate generalization in these tasks (*25*). We found that the modular agent exhibited accurate belief and robust control in these unencountered tasks, showcasing a capacity for instant generalization comparable to macaques’ ability in these tasks (*21*, *25*). In contrast, agents with less-specialized modules demonstrated inferior generalization performance. Furthermore, since there is no universal inductive bias that aids generalization across all tasks (the NFL theorem) (*13*), we also provided insights into why the modular agent’s knowledge acquired from training proves valuable for these unseen tasks, and when it does not.

## RESULTS

### RL agents trained to navigate using partial and noisy sensory cues

**Fig. 1.**
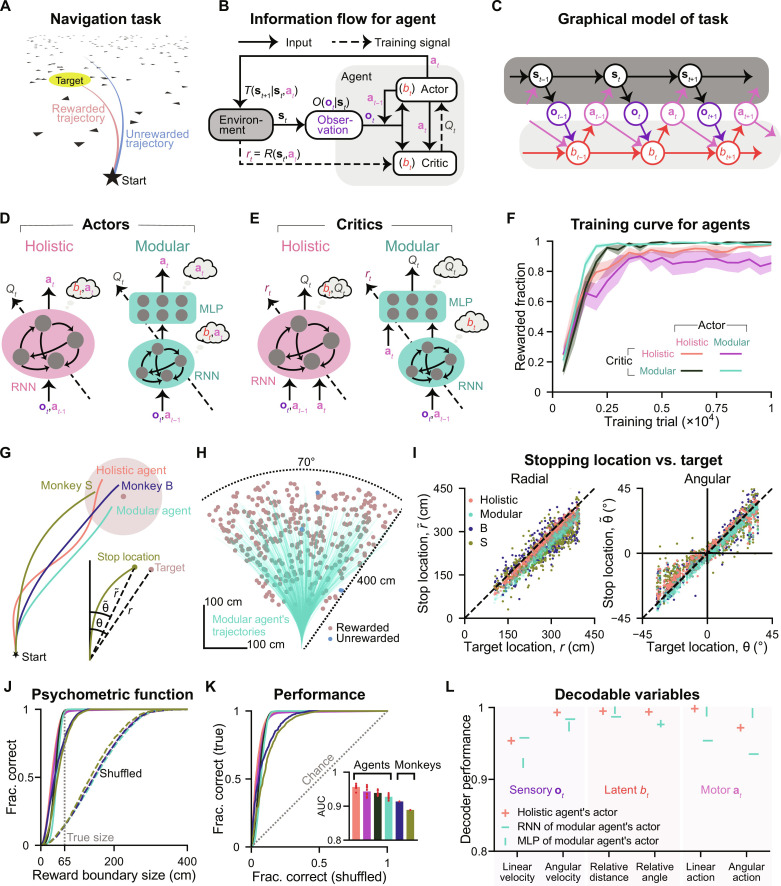
RL agents with different neural architectures were trained in a partially observable navigation task. (**A**) Schematic of the navigation task from the subject’s perspective. (**B**) Block diagram showing the interaction between an RL agent and the task environment. (**C**) Graphical model of the task. Environment update, dark gray; belief update, light gray. (**D**) Schematic of actors with a holistic (left) or modular (right) architecture. Thought bubbles denote the variables computed in each module. Dashed arrows indicate training signals. (**E**) Similar to (D) but for critic networks. (**F**) Fraction of rewarded trials during the training process following training phase 1, measured using a validation set (500 trials) for each agent at each checkpoint, which occurs every 500 training trials. Shaded regions denote ±1 SEM across training runs with eight random seeds. (**G**) An example trial showing monkeys and agents navigating toward the same target. Shaded circle, reward zone. Inset compares the target location versus the stop location of monkey S. (**H**) Overhead view of the spatial distribution of 500 representative targets and an example modular agent’s trajectories navigating toward these targets. (**I**) Comparison of agents/monkeys’ stop locations for the target locations from (H). Black dashed lines have a slope of 1. (**J**) Fraction of correct trials in a test set (1657 trials) as a function of hypothetical reward boundary size. Solid lines denote true data; dashed lines denote shuffled data. The gray dotted line denotes the true reward boundary size. (**K**) True data versus shuffled data in (J) (ROC curve). Inset shows AUC. (J) to (K) Agents’ data are averaged across eight training runs. (**L**) Performance (Pearson’s *r*) of linear decoders trained to decode task variables from example neural modules using trials in (J).

To study naturalistic, continuous time computations involved in foraging behaviors, we previously designed a virtual reality navigation task where macaques navigate to targets using sparse and transient visual cues (*20*). At the beginning of each trial, the subject is situated at the center of the ground plane (with a radius of 70 m) facing forward; a target is presented at a random location within the field of view (distance: 100 to 400 cm, angle: −35° to +35°) on the ground plane and disappears after 300 ms. The subject can freely control its linear and angular velocities with a joystick (maximum: 200 cm/s and 90°/s, referred to as the joystick gain) to move along its heading in the virtual environment (Fig. 1A). The objective is to navigate toward the memorized target location and then stop inside the reward zone—a circular region centered at the target location with a radius of 65 cm. A reward is given only if the subject stops inside the reward zone. The subject’s self-location is not directly observable because there are no stable landmarks; instead, the subject needs to use optic flow cues on the ground plane to perceive self-motion and perform path integration. Each textural element of the optic flow, an isosceles triangle with a base and a height of 8.5 and 18.5 cm, appears at random locations and orientations, disappearing after only a short lifetime (∼250 ms), making it impossible to use as a stable landmark. A new trial starts after the subject stops moving or the trial exceeds the maximum trial duration of 7 s. Given the target distance and joystick velocities, an optimal subject should reach the furthest target in around 2 s. Details of this task are described in (*20*). Macaques can be trained to master this task, and all macaque data presented in this paper were adapted from previously published works (*20*, *21*, *26*, *27*).

RL (*23*) is a reasonable framework for modeling behavior in this task because, like animals, RL agents can learn this task through sparse reward signals. We formulate this task as a partially observable Markov decision process (POMDP) (*28*) in discrete time, with continuous state and action spaces (Fig. 1B). At each time step *t*, the environment is in the state **s***_t_* (including the agent’s position and velocity and the target’s position). The agent takes an action **a***_t_* (controlling its linear and angular velocities) to update **s***_t_* to the next state **s**_*t*+1_ following the environmental dynamics given by the transition probability *T*(**s**_*t*+1_ ∣ **s***_t_*, **a***_t_*) and receives a reward *r_t_* from the environment following the reward function *R*(**s***_t_*, **a***_t_*) (a positive scalar if the agent stops inside the reward zone). The task transition probabilities are Markovian because **s**_*t*+1_ depends directly only on variables at *t* and is conditionally independent of all previous variables.

We use a model-free actor-critic approach to learning (Fig. 1B) (*23*), with the actor and critic implemented using distinct neural networks. At each *t*, the actor receives two sources of information about the state: observation **o***_t_* and last action **a**_*t*−1_. It then outputs an action **a***_t_*, aiming to maximize the state-action value *Q_t_*. This value is a function of the state and action, representing the expected discounted rewards when an action is taken at a state, and future rewards are then accumulated from *t* until the trial’s last step. Since the ground truth value is unknown, the critic is used to approximate the value. In addition to receiving the same inputs as the actor to infer the state, the critic also takes as inputs the action *a_t_* taken by the actor in this state. It then outputs the estimated *Q_t_* for this action, trained through the temporal-difference reward prediction error (TD error) after receiving the reward *r_t_* (∣*r_t_* + γ*Q*_*t*+1_ − *Q_t_*∣, where γ denotes the temporal discount factor). In practice, our algorithm incorporates additional mechanisms to enhance training (fig. S1, A and B; see Materials and Methods) (*29*).

The state *s_t_* is not fully observable, so the agent must maintain an internal state representation (belief *b_t_*) for deciding **a***_t_* and *Q_t_*. Both actor and critic undergo end-to-end training through backpropagation (BP) without explicit objectives for shaping *b_t_*. Consequently, networks are free to learn diverse forms of *b_t_* encoded in their neural activities that aid them in achieving their learning objectives. Ideally, networks may develop an effective Markovian belief update rule akin to recursive Bayesian estimation (although this is not guaranteed; Materials and Methods). Recursive Bayesian estimation infers a posterior density over the world state **s***_t_* from two primary sources of evidence. The first source involves predicting the state **s***_t_* based on its internal model of the dynamics, its previous posterior *b*_*t*−1_, and the last self-action **a**_*t*−1_ (e.g., a motor efference copy). The second source is a partial and noisy observation **o***_t_* of **s***_t_* drawn from the observation probability *O*(**o***_t_* ∣ **s***_t_*) (Fig. 1C). Note that the actual *O* in the brain for this task is unknown. For simplicity, we model **o***_t_* as a low-dimensional vector, including the target’s location when visible (the first 300 ms, Δ*t* = 0.1 s), and the agent’s observation of its velocities through optic flow, with velocities subject to Gaussian additive noise. Full details of this formulation are shown in Materials and Methods.

Actor and critic networks can have a variety of architectures. Our goal here is to investigate whether functionally specialized modules provide advantages for our task. Therefore, we designed architectures incorporating modules with distinct levels of specialization for comparison. The first architecture is a holistic actor/critic, comprising a single module where all neurons jointly compute the belief and the action/value. In contrast, the second architecture is a modular actor/critic, featuring modules specialized in computing different variables (Fig. 1, D and E). The specialization of each module is determined as follows. First, we can confine the computation of beliefs. Since computing beliefs about the evolving state requires integrating evidence over time, a network capable of computing belief must have some form of memory. Recurrent neural networks (RNNs) satisfy this requirement by using a hidden state that evolves over time. In contrast, computations of value and action do not need additional memory when the belief is provided, making memoryless multilayer perceptrons (MLPs) sufficient. Consequently, adopting an architecture with an RNN followed by a memoryless MLP [modular actor/critic in Fig. 1 (D and E)] ensures that the computation of belief is exclusively confined to the RNN. Second, we can confine the computation of the state-action value *Q_t_* for the critic. Since a critic is trained end-to-end to compute *Q_t_*, stacking two modules between all inputs and outputs does not limit the computation of *Q_t_* to a specific module. However, since *Q_t_* is a function of the action **a***_t_*, we can confine the computation of *Q_t_* to the second module of the modular critic (Fig. 1E, right) by supplying **a***_t_* only to the second module. This ensures that the first module, lacking access to the action, cannot accurately compute *Q_t_*. Therefore, the modular critic’s RNN is dedicated to computing *b_t_* and sends it to the MLP dedicated to computing *Q_t_*. This architecture enforces modularity and hierarchical computation.

For the modular actor (Fig. 1D, right), while we know that *b_t_* computation is confined to the RNN, there is no straightforward way to confine **a***_t_* computation to the MLP module through architecture design when both modules are trained end-to-end. Although a well-trained modular actor may learn sequential computation by computing **a***_t_* only in the MLP, it is not enforced, and **a***_t_* may still be distributively computed in both modules. Nevertheless, the modular actor has higher specialization than the holistic actor, which lacks confined *b_t_* computation. Thought bubbles in Fig. 1 (D and E) denote the variables that can be computed within each module enforced through architecture rather than indicating they are encoded in each module. For example, *b_t_* in modular architectures is passed to the second module, but an accurate *b_t_* can only be computed in the first RNN module.

We trained agents using all four combinations of these two actor and critic architectures (Fig. 1F, legend). We refer to an agent whose actor and critic are both holistic or both modular as a holistic agent or a modular agent, respectively. The training concluded after agents had experienced 10^4^ trials (after training phase 1; see Materials and Methods). Agents with modular critics demonstrated greater consistency across various random seeds (Fig. 1F, shaded regions) and achieved near-perfect accuracy more efficiently than agents with holistic critics.

Agents’ behavior was compared with that of two monkeys (Fig. 1G) for a representative set of targets uniformly sampled on the ground plane (modular/holistic agent; Fig. 1H and fig. S1C). In the next section, we will contrast the properties of agents’ trajectories, but first, we focus on the accuracy of their stop locations (linear: $\tilde{r}$ , angular: $\tilde{\theta }$ ) versus the target location (linear: *r*, angular: θ; Fig. 1G, inset). The tight correspondence between stop and target locations indicates that, similar to monkeys, all agents had learned the training task (Fig. 1I; Pearson’s *r*: fig. S1D). When stop locations were regressed against target locations (without intercept), we noticed that, similar to monkeys, agents also systematically undershot targets (fig. S1E; regression slope <1). This finding can be predicted based on the RL framework: Although the immediate reward for stopping at any location within the reward zone is the same, those considering long-term values discounted over time should prefer closer reward locations to save time.

We used a receiver operating characteristic (ROC) analysis (*20*, *21*) to systematically quantify behavioral performance. A psychometric curve for stopping accuracy is constructed from a large representative dataset by counting the fraction of rewarded trials as a function of a hypothetical reward boundary size (radius 65 cm is the true size; infinitely small/large reward boundary leads to no/all rewarded trials). A shuffled curve is constructed similarly after shuffling targets across trials (Fig. 1J). Then, an ROC curve is obtained by plotting the psychometric curve against the shuffled curve (Fig. 1K). An ROC curve with a slope of 1 denotes a chance level (true = shuffled) with the area under the curve (AUC) equal to 0.5. High AUC values indicate that all agents reached good accuracy after training (Fig. 1K, inset). This accuracy can be explained by accurate task variables encoded in their neural networks (actor: Fig. 1L, critic: fig. S1F; see Materials and Methods), as previously also shown in the macaque brain (*21*, *27*, *30*). Note that the modular agent achieved near-perfect accuracy (Fig. 1F); therefore, a slightly higher AUC for the holistic agent than for the modular agent (Fig. 1K, inset) does not imply that the holistic agent is more accurate. Instead, this small AUC difference arises because the modular agent is more optimal in stopping near the boundary of the reward zone, saving time while maintaining accuracy (see below).

### Different architectures, different beliefs and actions

After training, the critic no longer interacts with the environment; only the actor does. Therefore, agents’ behaviors rely on the two variables their actors compute: the internal belief and the action based on this belief. In this section, we investigate these two variables for all agents.

**Fig. 2.**
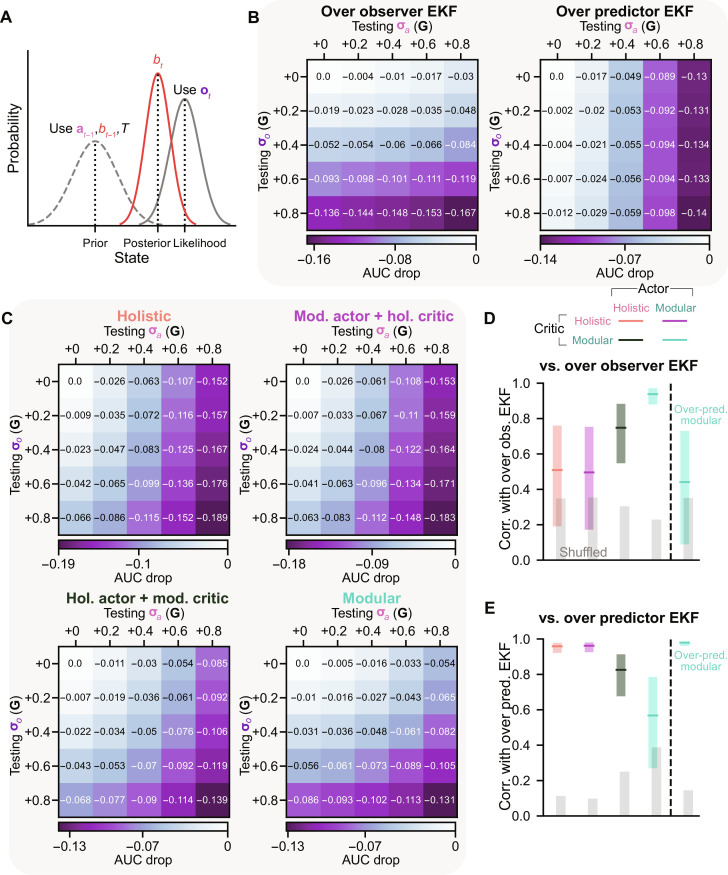
The modular agent learned an EKF-like belief. (**A**) Schematic of a Kalman filter in the 1D case, given zero-mean Gaussian noises. The prior is a prediction of the state **s***_t_* using the last action **a**_*t*−1_, the last belief *b*_*t*−1_, and the state transition *T*. The state’s likelihood depends on visual observation **o***_t_*. The posterior *b_t_* combines these two sources and provides a state estimation with an uncertainty smaller than only relying on a single source. (**B**) AUC drop with increased testing uncertainties from the training uncertainties, averaged across eight random seeds. We ran 2000 trials for each seed. The uncertainties for agent training and computing the Kalman gain in the EKF module were σ*_a_* = 0.2**G** and σ*_o_* = 0.1**G** for the over observer EKF (left) or were σ*_a_* = 0.1**G** and σ*_o_* = 0.2**G** for the over predictor EKF (right). We tested combinations of five levels of uncertainties: {+0, +0.2, +0.4, +0.6, +0.8}**G** higher than the training uncertainties for both σ*_a_* and σ*_o_*. For example, when the training uses σ*_a_* = 0.2**G**, then the tests use σ*_a_* = {0.2,0.4,0.6,0.8,1}**G**. (**C**) Similar to (B) but for agents using RNNs to construct beliefs. The training uncertainties are identical to those used for over observer EKF in (B). (**D**) Correlation between AUC drop of over observer EKF in (B) and AUC drop of other agents using RNNs to construct beliefs [those in (C) and fig. S2B]. Error bars denote 95% confidence intervals (CIs) obtained through bootstrapping. Gray, correlation of shuffled data. (**E**) Similar to (D) but using over predictor EKF instead of over observer EKF in (B) for computing correlations.

We first examine the belief. Using noisy observations **o***_t_* and noisy predictions from a motor efference copy **a**_*t*−1_, the optimal belief can be constructed through a Kalman filter (*24*). This filter implements recursive Bayesian estimation when all variables are Gaussian and the state transitions and observations are linear (Fig. 2A and Materials and Methods). It computes a posterior (belief) from a prior (based on its prediction) and likelihood (based on its observation) in two steps. In the prediction step, a prior for the current state is computed using the last self-action **a**_*t*−1_ and the last belief *b*_*t*−1_, following the state transition *T*. The predicted result has two uncertainty sources: one is the uncertainty in the last belief, denoted as *P*_*t*−1_; the other is the uncertainty of process noise associated with prediction, denoted as a covariance matrix Σ*_a_*. For our task, process noise only arises in the velocity given **a**_*t*−1_; the position is simply an integral of the velocities, so there is no additional process noise. The process noise in the linear and angular components is independent (Eq. 4 in Materials and Methods), so the only nonzero elements in Σ*_a_* are two variances $\sigma_{a_{v}}^{2}$ and $\sigma_{a_{\omega }}^{2}$ . These matrix elements correspond to SDs σ*_a_v__* and σ_*a*_ω__, which we put into a vector σ*_a_* and express in units of the linear and angular joystick gains σ*_a_* = α*_a_***G**. For example, when α*_a_* = 0.2 and **G** = [200 cm/s,90°/s]^⊤^ and then σ*_a_* = 0.2**G** = [40 cm/s,18°/s]^⊤^.

In the update step, the posterior (belief *b_t_*) multiplies the prior from the prediction and the likelihood given the observation **o***_t_*, leading to weighted combinations of the means and covariances. The weight on the observation is known as the Kalman gain, which gives greater weight to the source with smaller uncertainty. The observation noise covariance is denoted as Σ_o_, where the diagonal elements are the two variances $\sigma_{o_{v}}^{2}$ and $\sigma_{o_{\omega }}^{2}$ for the observation of linear and angular velocities (Eq. 5). All off-diagonal elements are zero. σ*_o_v__* and σ_*o*_ω__ can be put into a vector and are in units of **G**, i.e., σ*_o_* = α*_o_***G**.

Agents’ RNNs may learn a robust belief update rule akin to the Kalman filter [more precisely, an extended Kalman filter (EKF) (*31*) allowing nonlinear transitions]. In this scenario, during training, the RNN would infer σ*_a_* and σ*_o_* from its inputs **a**_*t*−1_ and **o***_t_* (in contrast to the EKF where these uncertainties are provided) and internalize an accurate Kalman gain based on these two uncertainties. Note that for our task, the Kalman gain relies solely on σ*_a_* and σ*_o_* and is independent of prior uncertainty *P*_*t*−1_ (Materials and Methods).

During the training in the last section, σ*_a_* = 0.2**G** and σ*_o_* = 0.1**G** for all agents, referred to as the default training uncertainties. If an agent develops an EKF-like belief, the learned Kalman gain should weigh more on observations (since σ*_o_* < σ*_a_*). Consequently, we hypothesize that in testing, the belief accuracy should be greatly affected if observations are less reliable than in training. Conversely, it should not be as greatly affected if predictions become more unreliable than in training.

To investigate this, we first defined an EKF agent (fig. S2A) that computes beliefs in its actor and critic using EKF modules instead of RNNs. We similarly trained this agent in the task using the default uncertainties. The EKF module requires uncertainties to be provided to compute the Kalman gain, which is then used to weigh prediction and observation. During training, ground truth uncertainties were provided for computing the Kalman gain. After training, during testing, we fixed the Kalman gain in the EKF module to be the same as that in training, but we increased the uncertainties in the task environment beyond the training uncertainties. The performance change with these testing uncertainties, measured by the AUC drop from the AUC with training uncertainties (Fig. 2B, left: “over observer EKF”), shows that the accuracy of this EKF belief, which has a Kalman gain that weighs observation more heavily, is less susceptible to increased σ*_a_* but more susceptible to increased σ*_o_*, consistent with our hypothesis. Conversely, when trained and using more reliable prediction than observation for computing its Kalman gain, e.g., σ*_a_* = 0.1**G** and σ*_o_* = 0.2**G** (reversed training uncertainties), an EKF agent has a belief that is less affected by increased σ*_o_* but more affected by increased σ*_a_* (Fig. 2B, right: “over predictor EKF”).

With these baselines established, we conducted similar tests by increasing uncertainties to levels higher than the training uncertainties for all trained agents using RNNs for beliefs (Fig. 2C). These agents were trained with the default training uncertainties, the same as that for over observer EKF. Agents using holistic critics exhibited performance changes more akin to over predictor EKF, while the modular agent aligned most closely with over observer EKF (Fig. 2, D and E). The modular agent resembled over predictor EKF only when trained with reversed uncertainties (“over predictor modular”; fig. S2B).

These results suggest that agents with different architectures learned to rely on different information sources. The modular agent’s belief closely aligns with the EKF, relying more on the source with smaller uncertainty. In contrast, agents using holistic critics weighed the less reliable source more heavily, and improving their belief requires either training data with a larger uncertainty difference between σ*_a_* and σ*_o_* (fig. S2, C and D) or a much longer training time (fig. S2, E and F). Nevertheless, although the holistic agent learned a belief that was suboptimal for the training task, this belief may be beneficial for other tasks, resulting in better performance than the modular agent in those tasks (fig. S2, G to I; see Discussion).

**Fig. 3.**
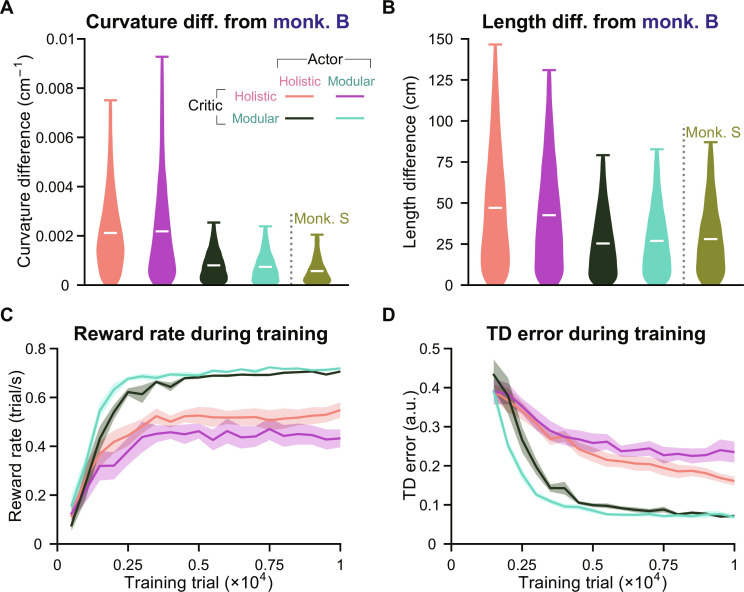
Agents with modular critics exhibit superior efficiency and performance in learning. (**A**) Distribution of the absolute curvature difference between agents’ and monkey B’s rewarded trajectories, as well as the difference between two monkeys’ rewarded trajectories, while all navigated to the same set of 1000 targets. The curvature values were averaged across time steps for each trajectory. (**B**) Similar to (A) but showing the absolute length difference for each trajectory. (A) and (B) Containing data from eight random seeds for each agent. White bars denote means across trials. (**C**) Similar to Fig. 1F but showing the reward rate. For each checkpoint, the reward rate is calculated by dividing the number of rewarded trials in a validation set (500 trials) by the time spent in seconds. (**D**) Similar to (C) but showing the agents’ TD error averaged across time steps and trials in the validation set after they reached an average accuracy of 60% across seeds. At each step *t*, the critic computes *Q_t_* based on the state and action at *t* and *Q*_*t*+1_ based on the state and action at *t* + 1. The TD error is then ∣*r_t_* + γ*Q*_*t*+1_ − *Q_t_*∣, where *r_t_* and γ denote the current reward and the discount factor (Materials and Methods). a.u., arbitrary units.

Next, we investigate agents’ actions. While we demonstrated in the last section that all agents’ stop locations were accurate after training (Fig. 1K), we also noticed distinct characteristics in their trajectories (Fig. 1G). To quantify these differences, we examined two crucial trajectory properties: curvature and length. When tested on the same series of targets as the monkeys experienced, agents with modular critics displayed more efficient trajectories than those with holistic critics, characterized by smaller curvature and length (fig. S3, A and B). Notably, the difference between trajectories generated by agents with modular critics and those of monkey B was comparable to the variation between trajectories of two monkeys (Fig. 3, A and B). In contrast, when agents used holistic critics, the difference in trajectories from monkey B was much larger, suggesting that modular critics facilitated more animal-like behaviors.

Agents are expected to develop efficient behaviors, as the value of their actions gets discounted over time. Therefore, we assess their efficiency throughout the training process by measuring the reward rate, which refers to the number of rewarded trials per second. We found that agents with modular critics achieved much higher reward rates (Fig. 3C), which explains their more efficient trajectories (fig. S3, A and B).

Since the actors responsible for generating actions were trained by maximizing the critics’ value estimation instead of the latent ground truth value, the lower reward rates may be attributed to inaccurate value estimation. To investigate this, we monitored the TD error for critics during training. This error is the discrepancy between the current value estimate and the discounted subsequent value estimate combined with the current reward, serving as the learning objective for the critic (Materials and Methods and Fig. 3D) (*23*, *29*). A critic that perfectly comprehends the task dynamics and rewards should yield no errors. Agents with modular critics exhibited faster convergence of TD errors, ultimately reaching much lower values compared to agents with holistic critics (Fig. 3D). This suggests that the modular critic enhances efficiency and accuracy in learning the task structure, thereby providing a training signal that closely aligns with the true nature of the task for the actor. Consequently, actors can develop superior behavior in the training task, as opposed to those trained by the holistic critic (Fig. 3C).

Furthermore, it is worth noting that the efficacy of actions depends on the quality of the beliefs in the actor. Inferior actions by agents using holistic critics may arise from accurate action choices coupled with inaccurate beliefs. To investigate whether this action itself is worse despite the quality of belief, we introduced a “holistic EKF” agent incorporating a holistic critic and an EKF actor (fig. S3C). Despite conditioning the control on a perfect EKF belief, this agent still fell short when compared to a modular agent in terms of trajectory characteristics and reward rate (fig. S3, D to F). This deficiency was attributed to the inferior training signal from the holistic critic for the action (fig. S3G).

Together, these results suggest that modular critics provide a superior training signal compared to holistic critics, allowing actors to learn more optimal beliefs and actions. With a poor training signal from the holistic critic, the modularization of actors may not enhance performance. Next, we will evaluate the generalization capabilities of the trained agents to understand how the quality of belief and action influences generalization.

### Gain task: Generalization to previously unencountered sensorimotor mappings

One crucial aspect of sensorimotor mapping is the joystick gain, which linearly maps motor actions on the joystick (dimensionless, bounded in [−1,1]) to corresponding velocities in the environment. During training, the gain remains fixed at 200 cm/s and 90°/s for linear and angular components, referred to as the 1× gain. By increasing the gain to values that were not previously experienced, we create a “gain task” manipulation. Monkeys demonstrated immediate generalization to unencountered gains and other task manipulations (*25*). This prompts us to investigate whether our trained agents can demonstrate similar generalization abilities. One distinction between animals and agents in unencountered tasks is that monkeys faced no constraints in learning, while the neural weights in our agents were frozen. Nevertheless, monkeys’ performance was stable in this gain task since the first trial (*25*), indicating that they do not rely on lengthy learning to grasp these novelties (see Discussion).

**Fig. 4.**
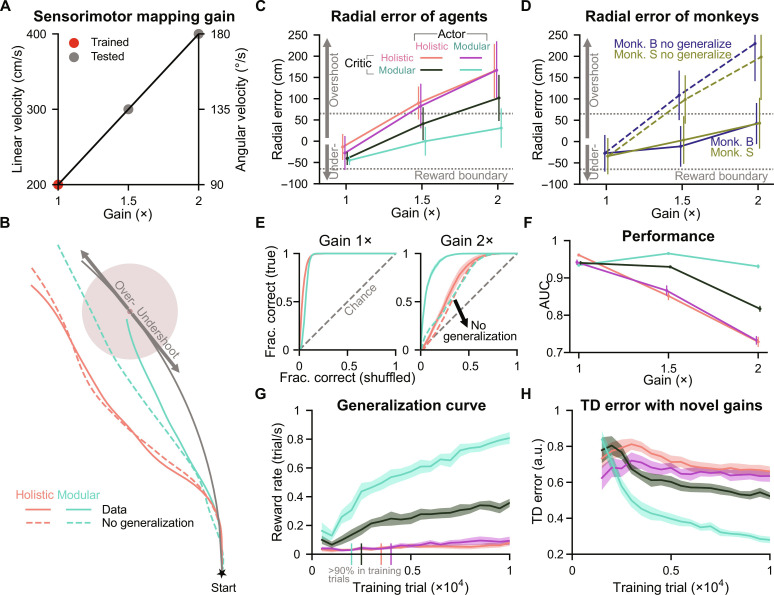
Modular agent exhibits the best generalization performance in the gain task. (**A**) Gain is the parameter that linearly maps the joystick actions onto velocities in the environment. The 1× gain used in training has linear and angular components of 200 cm/s and 90°/s, respectively. After training, 1.5× and 2× gains were used for testing. (**B**) Example trajectories of agents navigating toward a target with an unencountered 1.5× gain. Dashed lines, hypothetical no generalization trajectories; arrows, regions of over- or undershooting relative to the distance along an idealized circular trajectory connecting the start location to the target (gray line). (**C**) Radial error of agents’ stop locations across different gains. For each gain and agent, 8000 trials were conducted by concatenating results from eight random seeds, with the same set of 1000 targets for each seed. Error bars denote ±1 SD across trials. The gray dotted lines denote the reward boundary of 65 cm, the same as in training. (**D**) Similar to (C) but for monkeys [the same 1000 targets as in (C) for each gain and monkey]. (**E**) ROC curves for agents in different gains. Gray dashed lines, the chance level; dashed lines in other colors, agents’ hypothetical no generalization hypotheses. (**F**) AUC for ROC curves for all agents and gain conditions. (**G** and **H**) Similar to Fig. 3 (C and D), but data were averaged over two validation sets (the same set of 500 targets, gain = 1.5× and 2×). Vertical bars overlaid on the *x* axis in (G) denote the first time agents reached 90% accuracy in the 1× validation set (the same 500 targets, averaged across seeds). (E) to (H) Lines denote means across eight random seeds for each agent; shaded regions or error bars denote ±1 SEM.

To assess generalization abilities, monkeys and agents (the “Agent selection” section in Materials and Methods) were tested with unencountered gains of 1.5× and 2× (Fig. 4A). Blindly following the same action sequence as in the training task would cause the agents to overshoot (no generalization hypothesis: Fig. 4B, dashed lines; Materials and Methods). Instead, the agents displayed varying degrees of adaptive behavior (Fig. 4B, solid lines). To quantitatively evaluate behavioral accuracy while also considering over/undershooting effects, we defined radial error as the Euclidean distance between the stop and target locations in each trial, with positive/negative sign denoting over/undershooting (using idealized trajectories, see Materials and Methods). Under the previously unencountered gains, agents with modular critics consistently exhibited smaller radial errors than agents with holistic critics (Fig. 4C), with the modular agent demonstrating the smallest errors, comparable to those observed in monkeys (Fig. 4D and fig. S4A). ROC analyses further confirmed the performance differences among agents (Fig. 4, E and F).

Similar to the assessment of the agents’ trajectory characteristics, reward rates, and TD errors in the previous section, we again evaluated these quantities, this time under the unencountered gains. Agents with modular critics displayed more animal-like generalization trajectories, higher reward rates, and lower TD errors than agents with holistic critics, with the modular agent showing the closest resemblance to monkeys, the highest reward rates, and the lowest TD errors (fig. S4, B and C, and Fig. 4, G and H). Notably, the modular agent not only learned the training task the fastest (Fig. 4G, vertical bars on the *x* axis) but also learned to generalize better and faster than other agents, continuing to improve its generalization with additional training trials (Fig. 4G). This trend is also evident in the accuracy of the agents’ value estimates on unencountered gain trials (Fig. 4H).

Together, these results demonstrate the impact of different inductive biases on generalization to unencountered gains. The modular critic enables better generalization than the holistic critic, and the combination of a modular critic and modular actor produces the best generalization performance.

### Generalization in the gain task, facilitated by agents’ belief accuracy

Although we have confirmed that agents with distinct neural architectures exhibit varying levels of generalization in the gain task, the underlying mechanism remains unclear. We hypothesized that agents with superior generalization abilities should generate actions based on more accurate internal beliefs within their actor networks. Therefore, the goals of this section are to quantify the accuracy of beliefs across agents tested on unencountered gains and to examine the impact of this accuracy on their generalization performance.

**Fig. 5.**
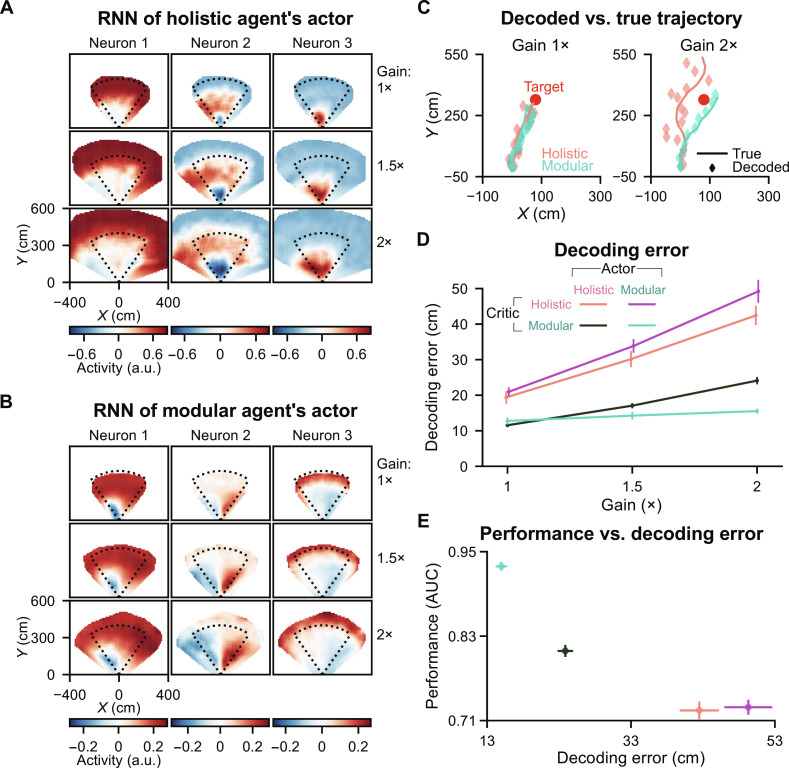
Decoding error of agents’ internal beliefs correlates with their behavioral performance in the gain task. (**A**) Spatial tuning of example RNN neurons in a holistic agent’s actor. Each column denotes a neuron; each row denotes a gain condition (2000 trials). Dotted lines denote the boundary of a region containing all target locations. (**B**) Similar to (A) but for example RNN neurons in a modular agent’s actor. (**C**) Decoded belief trajectories versus agents’ true trajectories during navigation to an example target under different gain conditions. Agents’ belief trajectories were estimated by linear decoders trained to decode agents’ locations from RNNs’ activities in actors (Materials and Methods). (**D**) Decoding error as a function of gain. This error is defined as the distance between the true and decoded locations at each time step and is averaged across time steps and trials in the test set. (C) and (D) For each seed of each agent in each gain condition, 1400 trials were used to train a decoder, and another 600 trials were used for analyses. (**E**) AUC versus decoding error for 2× gain. For each seed of each agent, 3500 trials were used to train a decoder, and another 1500 trials were used for analyses. (D) and (E) Error bars denote ±1 SEM across eight random seeds.

During the gain task, we recorded the activities of RNN neurons in the agents’ actors, as these neurons are responsible for computing the beliefs that underlie actions (Fig. 1D). As expected, these neurons showed sensitivity to the agents’ locations within the environment (spatial tuning; holistic agent: Fig. 5A, modular agent: Fig. 5B; Materials and Methods). To systematically quantify the accuracy of these beliefs, we used linear regression (with 𝓁_2_ regularization) to decode agents’ locations from the recorded RNN activities for each gain condition (Fig. 5C; Materials and Methods). We defined the decoding error, which represents the Euclidean distance between the true and decoded locations, as an indicator of belief accuracy. While all agents demonstrated small decoding errors under the training gain, we found that agents struggling with generalization under increased gains (Fig. 4F) also displayed reduced accuracy in determining their own location (Fig. 5D and fig. S5A). Agents’ behavioral performance correlates with their belief accuracy (trial-average: Fig. 5E; trial-by-trial: fig. S5B), a trend that was also observed in monkeys (*21*).

In Fig. 2, we demonstrated that different architectures yield distinct belief update rules after training, with the modular agent’s belief resembling an EKF. Our analyses here further indicate that this EKF-like belief enables a more accurate state representation in previously unencountered gains, leading to superior generalization.

### Perturbation task: Generalization to passive motions, facilitated by belief accuracy

**Fig. 6.**
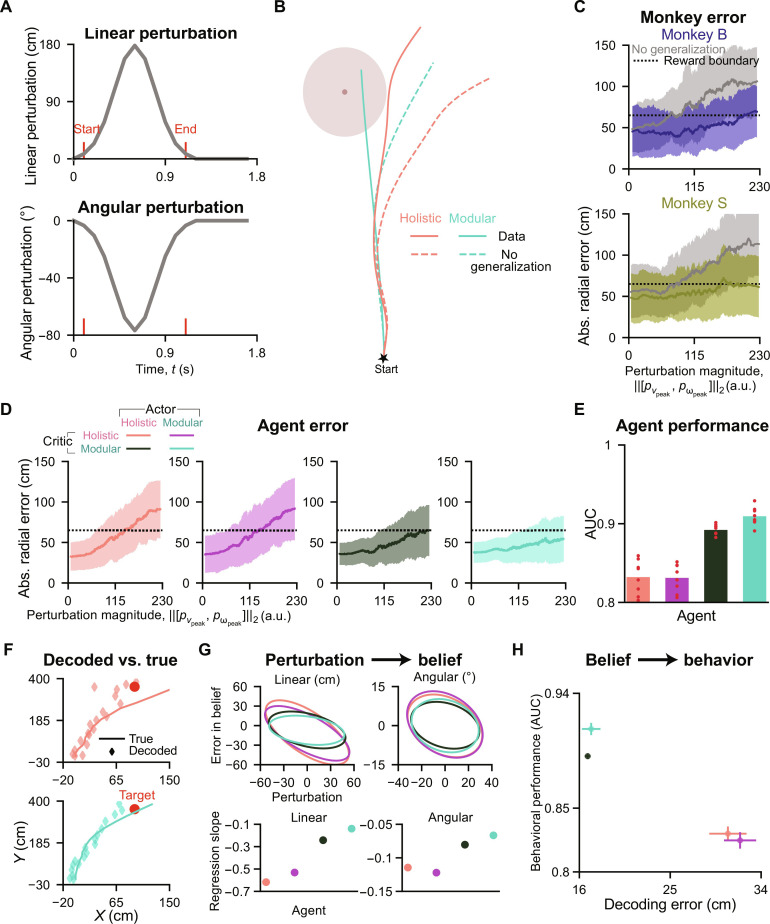
Agents with modular critics exhibit better generalization performance and more accurate internal beliefs in the perturbation task. (**A**) An example of sampled linear and angular perturbations in a trial. (**B**) Agents navigate in an example trial under the perturbations shown in (A). Dashed lines denote hypothetical no generalization trajectories. (**C**) Monkeys’ absolute radial error as a function of perturbation magnitude (the Euclidean norm of linear and angular perturbations) in 1000 trials. Gray, hypothetical no generalization trajectories. Solid lines and shaded regions denote means and ±1 SD obtained using a moving window (size = 100 trials). The dotted black line denotes the reward boundary of 65 cm, as in training trials. (**D**) Similar to (C) but for agents [1000 trials with target locations and perturbation parameters identical to those in (C) for each seed of each agent, resulting in 8000 trials for each agent; moving window size=800 trials]. (**E**) AUC for data with perturbation magnitude greater than 115 in (D). Bars denote means across random seeds; red dots denote data for individual seeds. (**F**) Similar to Fig. 5C but showing trajectories navigating to an example target under perturbation. (**G**) Top: Confidence ellipses for the deviation of decoded stop locations from true stop locations (left/right: using locations’ linear distance/polar angle) versus the integral of perturbation velocities (left/right: linear/angular perturbation) across trials. Bottom: Regression slope (without intercept) for the data. (**H**) Similar to Fig. 5E but for perturbation trials. (F) to (H) For each seed of each agent, 3500 trials were used to train a decoder, and another 1500 trials were used for analyses. The perturbation parameters were sampled from the ranges used in (C) and (D), but smaller linear (within [−100,100] cm/s) and angular ([−60,60]°/s) perturbations were excluded in (H).

To assess one’s ability for generalization with manipulated latent states in the environment, we introduce another task called the “perturbation task” (Materials and Methods). This task involves applying passive perturbation velocities to the joystick control at a random time for both the linear and angular components, causing monkeys or trained agents (the “Agent selection” section in Materials and Methods) to deviate from their intended trajectories. The perturbations follow a Gaussian temporal profile lasting for 1 s (Fig. 6A), with perturbation peak time relative to the trial start uniformly sampled in the range [0.5,1.5] second and peak magnitudes for the passive linear and angular velocities sampled in [−200,200] cm/s and [−120,120]°/s for each trial. Figure 6B illustrates an example trial, displaying agents’ adaptive behaviors in response to the sampled perturbations shown in Fig. 6A. If agents blindly follow the same action sequence as in the training task (Fig. 6B, no generalization hypothesis; see Materials and Methods), then they would deviate toward the perturbation direction.

Monkeys displayed adaptation to perturbations, as evidenced by their behavioral errors (Fig. 6C). When faced with the same perturbations, agents with modular critics displayed errors comparable to those of monkeys, which were much smaller than the errors produced by agents with holistic critics (Fig. 6D). ROC analysis also supports these findings (Fig. 6E). This performance difference can be attributed to the agents’ ability to adjust behaviors: Agents with modular critics demonstrated greater compensation for perturbations compared to those with holistic critics (fig. S6, A and B). Macaques and humans also exhibited compensatory behaviors in this task (*26*).

Similar to our observations in the gain task (Fig. 4, G and H), agents’ generalization abilities (measured by reward rates) under perturbation improved with increased exposure to training trials (fig. S6C), and those with higher reward rates demonstrated a better understanding of the perturbation task, as indicated by their lower TD errors (fig. S6D).

We further investigated the neural mechanisms underlying agents’ different generalization abilities. As agents’ locations were perturbed, their internal beliefs should continuously track these perturbed locations. Failure to do so would introduce errors into their internal beliefs, ultimately affecting generalization behaviors. To test this, similar to our approach in the gain task (Fig. 5), we recorded the agents’ locations and the activities of RNN neurons in their actors under perturbations. We then linearly decoded the agents’ locations from these activities (Fig. 6F; see Materials and Methods) and measured the difference between the true and decoded locations as an indicator of belief accuracy. We found that increased perturbations caused agents’ beliefs to deviate from the true locations (Fig. 6G, top, and fig. S6, E and F), with agents using modular critics being less affected than those using holistic critics (Fig. 6G, bottom). These belief errors, akin to what we observed in the gain task (Fig. 5E and fig. S5B) then propagated to behavioral errors (trial-average: Fig. 6H; trial-by-trial: fig. S6G).

Note that confidence ellipses used in the top two panels in Fig. 6G represent the bivariate distribution of data (assuming Gaussianity) in fig. S6 (E and F), produced by agents with eight random seeds each. The center of an ellipse denotes the mean, and the region inside indicates within 1 SD. A positive/negative tilt in the major axis of the ellipse indicates a positive/negative correlation, and a more circular ellipse suggests a correlation closer to zero (Pearson’s *r* in the same agent order as in Fig. 6G for the left: −0.67, −0.69, −0.42, and −0.30; for the right: −0.30, −0.31, −0.26, and −0.19; *P* = 0).

These analyses again demonstrate the impact of architectural inductive biases on generalization. By enabling actors to learn EKF-like beliefs that remain accurate under perturbations, modular critics facilitate superior generalization than holistic critics.

### Generalization contingent on learned Kalman gain

We have demonstrated that the modular agent learned an EKF-like belief (Fig. 2, D and E). This proved to be more accurate when encountering unseen gains and perturbations, facilitating generalization (Figs. 5E and 6H). Nevertheless, the reasons behind the high accuracy of an EKF-like belief in these tasks remain unclear.

Agents were trained with the default training uncertainty σ*_a_* = 0.2**G** in prediction and σ*_o_* = 0.1**G** in observation. Therefore, an EKF-like belief relies more on observations. Our gain and perturbation tasks have structures that also necessitate a greater reliance on observation: Subjects must be aware of novel gains or perturbations via observation of optic flow, as their internal model for prediction becomes outdated in these tasks. We hypothesize that the EKF-like belief in previous sections favors generalization because the training uncertainty aligns with the structure of unencountered tasks.

**Fig. 7.**
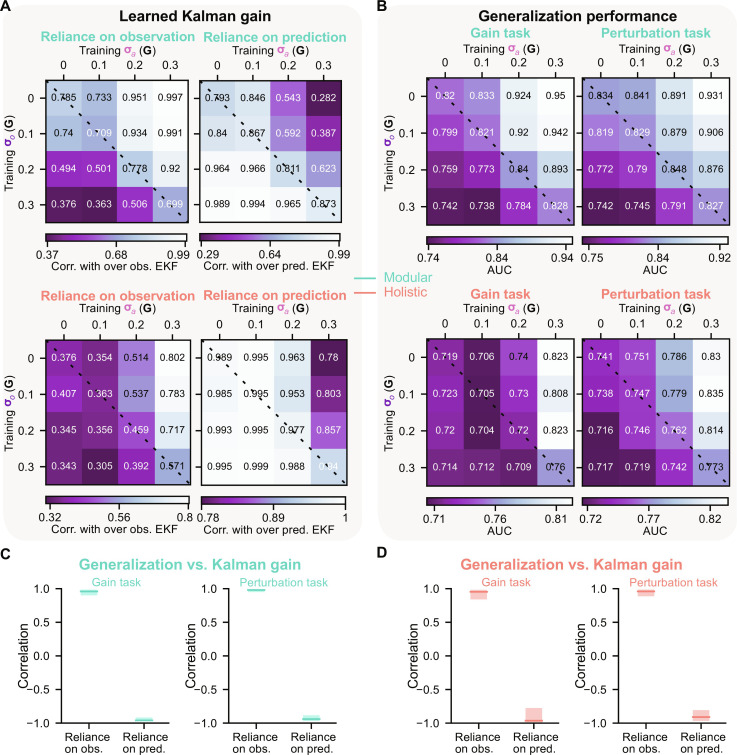
Generalization in gain and perturbation tasks requires learning to rely more on observation. (**A**) Left: Similar to Fig. 2D but showing modular (top) and holistic (bottom) agents trained with 16 combinations of σ*_a_* and σ*_o_*. Specifically, for each agent trained under a particular uncertainty condition, the AUC drop when facing testing larger uncertainties than seen in training was evaluated (similar to Fig. 2C). Subsequently, we measured the correlation between this AUC drop and that of the over observer EKF in Fig. 2B (left) (similar to Fig. 2D). Right: Similar to left but showing the correlation between the AUC drop of each agent and that of over predictor EKF in Fig. 2B (right) (similar to Fig. 2E). These two steps were repeated for each training uncertainty condition. (**B**) AUC for agents in (A) tested in the gain (left) and perturbation (right) tasks. In the gain task, the testing gain was 2×. In the perturbation task, the testing perturbation peak time was 0.5 s, and the peaks of perturbation linear and angular velocities were sampled uniformly from the ranges used in Fig. 6H. (A) and (B) Two thousand trials were used for each seed of each agent. Values for each agent were averaged across eight random seeds. Dashed lines denote when σ*_o_* = σ*_a_*. White/black text indicates below/above-average values. (**C**) Left: Correlation between modular agents’ learned Kalman gain [values in (A), top] and their generalization performance in the gain task [(B), top left ]. Right: Similar to left but using their generalization performance in the perturbation task [(B), top right]. (**D**) Similar to (C) but showing the holistic agent [(A), bottom and (B), bottom]. (C) and (D) Error bars denote a 95% CI obtained through bootstrapping.

To verify this, it is essential to train agents with various pairs of σ*_a_* and σ*_o_* so that for each pair, an EKF-like belief learns a unique reliance on observation, i.e., the Kalman gain. We trained 16 modular and 16 holistic agents, with each architecture being trained using a combination of σ*_a_* and σ*_o_* within {0,0.1**G**,0.2**G**,0.3**G**}. An uncertainty of 0 denotes the noise-free case. To investigate the Kalman gain learned by each agent, we conducted analyses (Fig. 7A, top: modular, bottom: holistic) similar to those in Fig. 2 (C to E). Specifically, just like in Fig. 2C, each agent trained under a unique uncertainty condition was tested with combinations of five levels of uncertainties {+0,+0.2,+0.4,+0.6,+0.8}**G** higher than the training uncertainties for both σ*_a_* and σ*_o_*. The resulting AUC drop table was then correlated with that of over observer EKF and over predictor EKF in Fig. 2B, a process akin to Fig. 2 (D and E). These two steps were repeated for all agents trained with all combinations of σ*_a_* and σ*_o_*, and these results are shown in Fig. 7A (left: correlation with over observer EKF, right: with over predictor EKF). A high correlation with over observer EKF indicates the belief’s high reliance on observation, while a high correlation with over predictor EKF indicates a high reliance on prediction.

Modular agents’ beliefs aligned with the latent uncertainties during training, relying more on observations when trained with σ*_o_* < σ*_a_* and leaning more toward prediction when trained with σ*_a_* < σ*_o_* (Fig. 7A, top). However, holistic agents had inferior abilities in learning beliefs that correspond to training uncertainties (Fig. 7A, bottom). This deficiency is evident in specific uncertainty conditions where observation was more reliable, yet holistic agents relied more on prediction.

Following the estimation of agents’ learned Kalman gains, we then assessed their performance in gain and perturbation tasks (see the “Agent selection” section in Materials and Methods). Modular agents (Fig. 7B, top) trained with smaller observation uncertainties (σ*_o_* < σ*_a_*) generalized better than those trained with equal uncertainties (σ*_o_* = σ*_a_*) and, worst of all, those trained with larger observation uncertainties (σ*_o_* > σ*_a_*). However, holistic agents generalized poorly in some uncertainty conditions where σ*_o_* < σ*_a_* (Fig. 7B, bottom). By comparing agents’ learned Kalman gains (Fig. 7A) and performance (Fig. 7B), generalization had a strong positive correlation with reliance on observation and, conversely, had a negative correlation with reliance on prediction (modular: Fig. 7C, holistic: Fig. 7D).

These findings confirm our hypothesis that constructing beliefs based more on observation aligns with the structure of our gain and perturbation tasks, so belief remains relatively accurate in these tasks and facilitates generalization. Learning to rely more on observation necessitates σ*_o_* < σ*_a_* during training. Modular agents can efficiently discern the more reliable input source for their beliefs, while holistic agents perform poorly in achieving this, leading to a reliance on prediction even when observation is more reliable in some conditions. With its inferior inductive bias for our tasks, holistic agents required a much extended training period (10^5^ trials after training phase 1, 10 times the previously used duration; Materials and Methods and fig. S7A) to learn the Kalman gains that match training uncertainties. As expected, this improvement in belief is associated with improved performance in the unencountered tasks (fig. S7B). Nevertheless, even after extensive training, holistic agents still generalized worse than modular agents (fig. S7B). This performance difference is more obvious when we evaluated them under more challenging gain and perturbation parameters (fig. S7C). Note that with equal uncertainties, the performance of agents trained without any noise (σ*_o_* = σ*_a_* = 0) is worse than that of agents trained with noise (σ*_o_* = σ*_a_* > 0; fig. S7C). This may suggest that noise injected into inputs can improve the robustness of learned solutions (*32*). Nevertheless, it is still the relative reliability between two uncertainties that plays a major role in shaping generalization.

We then investigate why the modular and holistic agents perform differently (fig. S7C) even when the holistic agent’s learned Kalman gain was accurate due to extensive training (fig. S7A). In Fig. 3, we demonstrated that besides beliefs, the holistic agent also developed inferior control actions. Here, we evaluated actions for agents extensively trained with the default uncertainty (σ*_a_* = 0.2**G**, σ*_o_* = 0.1**G**) under challenging gain and perturbation parameters (fig. S8, A and B). We also considered a holistic EKF agent, incorporating a holistic critic and an EKF actor (fig. S3C). We found that the holistic EKF agent developed more efficient actions and generalized better than the holistic agent, suggesting that actions are better when based on an accurate EKF belief. Nevertheless, the modular agent’s actions and performance were still superior to those of both the holistic EKF and EKF agents (fig. S8, A and B).

Together, these findings suggest that the modular agent has a better inductive bias than the holistic agent for our gain and perturbation tasks, resulting in much better data efficiency for acquiring the underlying structure of the training task. The modular architecture favors generalization when its learned knowledge aligns with the structure of unseen tasks. While additional training can ameliorate the shortcomings of the holistic architecture to some extent, it cannot entirely offset its inherent limitations.

### More architectures using less specialized modules

**Fig. 8.**
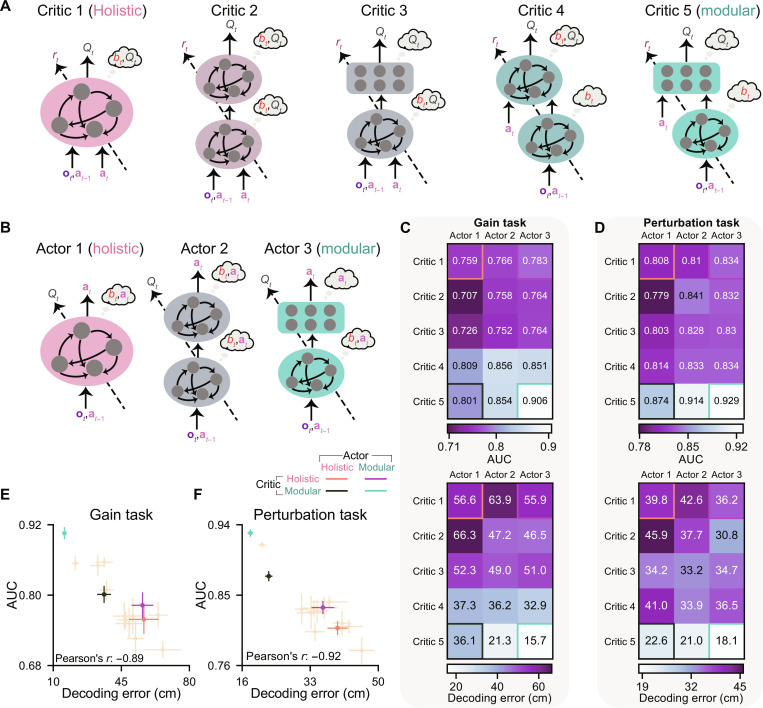
Agents using less specialized modules exhibit less accurate internal beliefs, resulting in inferior performance compared to the modular agent. (**A** and **B**) Critic and actor diagrams as in Fig. 1 (E and D) but including more architectures with less specialized modules. (**C** and **D**) AUC (top) and decoding error (bottom, averaged across time steps and trials) of agents in the gain (C) and perturbation (D) tasks, averaged across eight random seeds. For each seed of each agent, 2000 trials were conducted. Gains were sampled from [3×, 4×] for (C). Perturbation peak time was sampled from [0.5,1.5] s, and peaks of linear and angular perturbation velocities were sampled from [−200,800] cm/s and [−180,180]°/s for (D). Beliefs were decoded from the RNN for actors 1 and 3 or the first RNN for actor 2. The four corners represent the four agents used in the previous analyses. Text in white/black denotes that the agent is worse/better than the average value of all agents. (**E** and **F**) AUC versus decoding error using data in (C) and (D). Error bars denote ±1 SEM across random seeds.

Above, we compared the holistic architecture against the modular architecture to investigate the advantages of module specialization. In this section, we aim to further strengthen our argument by introducing more architectures that deviate from the modular architecture (Fig. 8, A and B).

By using two sequential RNNs instead of one, critic/actor 2 can distribute computations of two variables across two modules without enforced specialization. Substituting an MLP for the second RNN in critic/actor 2 yields critic/actor 3, where the belief computation over time is confined to the first RNN. Alternatively, we can retain two RNNs in the critic and exclusively provide the action input to the second RNN. This yields critic 4, where the value computation is confined to the second module. The modular critic confines both the belief and value computation and has the most module specialization. Note that the total number of trainable parameters is designed to be similar across all architectures (fig. S9A and see Materials and Methods).

We extensively trained agents using all combinations of these critics and actors (10^5^ trials) with default uncertainties σ*_a_* = 0.2**G** and σ*_o_* = 0.1**G**. We found that agents with less specialized critics still demonstrated lower reward rates than those with a modular critic (fig. S9B, left). This can be attributed to the less accurate value estimates provided by their critics for training their actors (TD errors; fig. S9B, right; Pearson’s *r* = −0.93).

We then compared these agents’ (the “Agent selection” section in Materials and Methods) generalization abilities (Fig. 8, C and D). Across critics, the modular critic (critic 5) outperformed all others. Note that the architectural components used in the pairs of critics 5 and 3 (RNN + MLP) and critics 4 and 2 (RNN + RNN) are identical. The only difference is that in critics 5 and 4, the value computation is confined to the second module, as only there can the computation access the action **a***_t_*, whereas critics 3 and 2 allow both modules to access the action, eliminating such confinement. The performance difference within each pair highlights the advantages of specialization for value computation. Similarly, in critic 5, the belief computation is confined to the first module, while in critic 4, it is not confined. The performance difference between critics 5 and 4 demonstrates that specialization for belief computation could further enhance performance. However, specializing in belief without specializing in value did not provide benefits, as indicated by the difference between critics 3 and 2 (mean AUC across random seeds: Fig. 8, C and D, top; SEM of AUC across random seeds: fig. S9C; reward rate: fig. S9D).

Actors’ performance was contingent on the choice of critic architectures, as actors were trained by critics to learn the task structure. With a modular critic, the modular actor benefits from a dedicated belief module, enabling the development of an internal belief that remains accurate in previously unseen tasks (Fig. 8, C and D, bottom). Consequently, it outperformed others with less accurate beliefs (Fig. 8, E and F).

Together, we conclude that the architecture using both the modular critic and modular actor represents the most appropriate inductive bias for our task, benefiting learning and generalization. The specialization among modules, rather than simply the number of modules, matters. To illustrate, critic 3 shares the same number and type of modules as critic 5, and it has more modules than critic 1. Nevertheless, critic 3 did not outperform critic 1 and performed worse than critic 5.

## DISCUSSION

The brain has evolved advantageous modular architectures for mastering daily tasks. Here, we investigated the impact of architectural inductive biases on learning and generalization using deep RL agents. We posited that an architecture with functionally specialized modules would allow agents to more efficiently learn essential task variables and their dependencies during training and then use this knowledge to support generalization in unseen tasks with a similar structure. To test this, we trained agents with architectures featuring distinct module specializations on a partially observable navigation task. We found that the agent using a modular architecture exhibited superior learning of belief and control actions compared to agents with weaker modular specialization. The modular agent’s beliefs are akin to an EKF, properly weighing information sources according to their relative reliability. Its actions are also more efficient and resemble trajectories of well-trained monkeys.

During our training task, the environment exhibits higher prediction uncertainty than observation uncertainty, so the EKF-like belief should—and does—rely more on observation than prediction. Subsequently, we tested trained agents in unseen tasks with parameters that also favor greater reliance on observation. In line with the parameters of these tasks, the EKF-like belief of the modular agent remains more accurate than the belief of alternative agents, facilitating better generalization in these tasks. Furthermore, the modular agent’s control is also more robust in handling these unseen tasks.

### Reasons for the benefits of modularization

One explanation of the benefits of modularization lies in its capacity to structure the underlying task variables and their relationships, allowing neural networks to learn the data-generating process more efficiently and facilitating generalization where the learned knowledge is reusable (*8*–*11*, *33*). This proves especially advantageous in natural tasks, where task variables typically exhibit sparse dependencies.

Inductive biases can be likened to “training data in disguise” (*8*). They offer useful priors that align more closely with the optimization solution, substantially reducing the search space and minimizing training time. Our findings demonstrated these principles. The holistic agent, lacking the inductive biases of modularization, needed substantially longer training than the modular agent to learn computations correctly weighing information sources (fig. S2F).

We designed the modular architecture (Fig. 1E, right) with priors of how latent variables are computed and their relationships. Specifically, the value computation in the critic involves observation **o***_t_*, previous action **a**_*t*−1_, current action **a***_t_*, and value *Q_t_*. This computation is broken down into two steps: firstly, computing *b_t_* in the belief module and then computing *Q_t_* in the value module based on *b_t_*. By design, the belief module receives **o***_t_* and **a**_*t*−1_ to compute *b_t_*, as *b_t_* should weigh these information sources. This module has recurrence since *b_t_* depends on *b*_*t*−1_. However, it does not consider **a***_t_* as an input, as **a***_t_* and *b_t_* are independent of each other. On the other hand, the value module operates by receiving the computed *b_t_* and **a***_t_* because *Q_t_* is dependent on these two. It does not have recurrence, as *Q_t_* is independent of *Q*_*t*−1_. The modular critic incorporates these variable relationships through architectural design. In contrast, the holistic critic lacks these priors embedded in its architecture and can only acquire these relationships through learning. However, the successful learning of these relationships is not assured, as agents with weaker module specialization exhibited inferior performance even after extensive training (fig. S9B).

It is also worth noting a key distinction from supervised learning: In training the critic, the learning target *r_t_* + γ*Q*_*t*+1_ is not fixed. The next value *Q*_*t*+1_ is bootstrapped by the critic itself or a delayed updated version (Materials and Methods) (*29*, *34*). Consequently, the learning target changes with updates of the critic, presenting an inherent challenge in achieving convergence (*34*). A strong inductive bias may effectively narrow down the solution search space, offering a more stable learning target. This is evident in the TD error (difference between the value estimation and the learning target) during training (Fig. 3D). This error for the modular critic converged faster and to a smaller error. A well-converged critic enhances the accuracy of its value estimates, serving as a more reliable training signal for the actor.

### Generalization but NFL

The NFL theorems proved that no inductive bias can excel across all tasks (*13*). When agents are evaluated in domains vastly distinct from their training settings, such as learning navigation but being tested in a bandit task, it is expected that generalization falters. Furthermore, even for tasks related to the training task, generalization may prove challenging if the acquired knowledge does not align with the structures of the new tasks.

Agents with a modular architecture can acquire the underlying structure of our training tasks. In contrast, holistic agents tend to acquire different knowledge from modular agents during training, such as forming beliefs based on unreliable information sources or exhibiting less efficient control actions. The gain and perturbation tasks have structures similar to the training task (when observations are more reliable), relying more on observation for belief formation and efficient steering. Consequently, a modular agent that accurately learns the training task’s structure can leverage its knowledge in these previously unseen tasks. However, it is worth noting that an infinite number of new tasks can be constructed, diverging from the training task’s structure but aligning with the “inferior” beliefs and control acquired by holistic agents.

Other tasks may be more aligned with the holistic agent’s belief. As in Fig. 2C, modifying uncertainties σ*_a_* and σ*_o_* to values higher than those during training can be regarded as an unencountered task. When this task presents much higher σ*_o_* than σ*_a_* (fig. S2, G and H, bottom left), the reliability of observations is considerably lower than that of predictions. Consequently, the holistic agent prioritizing predictions for belief outperformed the modular agent prioritizing observations in this scenario (fig. S2I).

A task can also be favored by the holistic agent’s control. During training, the reward for stopping at any location within the reward zone remains consistent and is discounted over time. The holistic agent tends to develop less efficient trajectories, stopping closer to the center of the reward zone, while the modular agent learned to stop nearer to the reward zone boundary to save time (fig. S1E), thereby achieving a higher reward rate (Fig. 3C). Introducing an unencountered task with a variable reward, decreasing as a function of the distance between the stopping location and the reward zone center, and not discounted over time, should result in superior performance by the holistic agent compared to the modular agent tested in this task.

### Limitation and future directions

Inductive biases play a crucial role in learning systems. However, when confronted with unseen tasks featuring structures distinct from those in training, intelligent systems, like animals, do not solely depend on existing knowledge. Instead, they also use efficient and adaptable learning algorithms to continually update their understanding of the new task at hand (*1*), a capability that our current models lack.

Various brain-inspired algorithms have been proposed to facilitate efficient learning of unseen tasks while leveraging previously acquired knowledge from old tasks. For example, the successor representation (*35*, *36*), replicating place and grid cell properties, decomposes the value into a representation of transition probabilities learned from training and a reward function model, allowing more efficient learning in previously unseen tasks with new rewards. By only learning rewards, values can be reconstructed using these rewards and previously learned transitions. Furthermore, the meta-RL algorithm (*37*), inspired by the standalone fast learning system of the prefrontal cortex that is shaped by (but distinct from) the slow dopamine-based RL (*38*), uses model-free values to train a standalone RNN policy network that maps inputs to actions across multiple tasks. This allows the policy network to learn not just a single policy but an embedded learning algorithm for learning new tasks.

Numerous previous studies have identified animal-like behavior and neural computation in RL agents in a diverse range of tasks (*35*, *37*, *39*–*43*). This “NeuroAI” paradigm (*44*) bridges neuroscience and AI, leveraging insights from the brain to enhance AI capabilities in tasks where animals excel naturally. A central challenge for this paradigm lies in reconciling various proposed brain-inspired algorithms, each advantageous for specific tasks. Addressing this challenge may involve incorporating modular inductive biases, recognizing the diverse functional and algorithmic specializations observed in different brain regions (*35*, *37*, *45*, *46*). Inspired by this and the advantages of modularization that we have presented, future investigations could develop large modular networks that integrate diverse algorithms proposed in previous studies (*35*, *37*, *39*–*43*) into distinct modules. For instance, we may equip our agent with efficient learning algorithms in new modules, enabling it to tackle previously unseen tasks with structures much different from its training data. Depending on the current task demands, agents could choose strategies such as zero-shot generalization, efficient learning to update specific knowledge aspects (*35*, *47*–*50*), or complete relearning.

It is worth noting that although our work compared the behaviors of animals and agents after training, their learning processes differ, and this difference is not the objective of our study (see Supplementary Text for details). Furthermore, future work may investigate the advantage of an inductive bias that enforces two different neural modules to approximate prediction and update steps in an EKF sequentially. Our current modular agent uses a single RNN to approximate the entire EKF computation (see Supplementary Text for details).

## MATERIALS AND METHODS

### Task

The navigation task and its manipulations were originally designed for macaques (*20*, *21*, *25*–*27*, *30*). All macaque data used in this paper were from previous works (*20*, *21*). Below, we provide a summary of the animal task setup based on (*20*).

Subjects used an analog joystick with two degrees of freedom to control their linear and angular speeds in a virtual environment. This virtual world comprised a ground plane with textural elements having a limited lifetime of ∼250 ms. The ground plane was circular with a radius of 70 m. The subject was positioned at its center at the beginning of each trial. Each texture element was an isosceles triangle (base by height: 8.5 cm by 18.5 cm) that randomly repositioned and reoriented anywhere in the arena. The maximum linear and angular speeds were fixed to 200 cm/s and 90°/s, respectively, and the density of the ground plane was either held constant at 2.5 elements/m^2^ or varied randomly between 0.1 and 2.5 elements/m^2^. The stimulus was rendered as a red-green anaglyph and projected onto the screen in front of the subject’s eyes. Subjects wore goggles fitted with Kodak Wratten filters to view the stimulus. The task was to steer to a random target location that was cued for 300 ms at the beginning of the trial. Each trial was programmed to start after a variable random delay between 0.5 and 1.1 s following the end of the last trial. The target was a circular disc with a radius of 20 cm, matching the luminance of the texture elements. It appeared at a random location between −35 and +35° of visual angle and at a distance of 100 to 400 cm relative to the subject at the beginning of the trial. Monkeys received a drop of juice if their stopping position was within 65 cm away from the center of the target. No juice was provided otherwise.

### Task modeling

We modeled this task as a POMDP (*28*) for RL agents, containing a state space *S*, an action space *A*, a transition probability *T*, a reward function *R*, an observation space Ω, an observation probability *O*, and a temporal discount factor γ = 0.97 over steps within a trial.

#### State

Each state **s***_t_* ∈ *S* is a vector [*s_x_t__*, *s_y_t__*, *s*_θ*_t_*_, *s_v_t__*, *s*_ω*_t_*_, *g_x_t__*, *g_y_t__*]^⊤^ containing the agent’s *x* and *y* positions (centimeters), head direction (degrees), linear and angular velocities (centimeter per second, degree per second), and the target’s *x* and *y* positions (centimeters). The initial state of each trial was defined as **s**_0_ = [0,0,90,0,0, *g*_*x*_0__, *g*_*y*_0__]^⊤^, since the agent always starts from the origin facing forward (90°). The target location was uniformly sampled on the ground plane before the agent, with the radius *g_r_* ∈ [100 cm,400 cm] and the angle *g*_θ_ ∈ [90° − 35°,90° + 35°] relative to the agent’s initial location. Specifically, angles were drawn uniformly within the field of view, *g*_θ_ ∼ 𝒰(55°,125°), and we sampled radial distances as $g_{r}∼\sqrt{U\left(100^{2},400^{2}\right)}$ to ensure a spatially uniform distribution in two dimensions (2D). Target positions in Cartesian coordinates are then *g*_*x*_0__ = *g_r_* cos (*g*_θ_), *g*_*y*_0__ = *g_r_* sin (*g_θ_*).

#### Action

Each action **a***_t_* ∈ *A* is a vector [*a_v_t__*, *a*_ω*_t_*_]^⊤^ containing the agent’s linear and angular joystick actions, bounded in [−1,1] for each component.

#### Transition

State transitions **s**_*t*+1_ ∼ *T*(**s**_*t*+1_ ∣ **s***_t_*, **a***_t_*) were defined as **s**_*t*+1_ = **f**_env_(**s***_t_*, **a***_t_*) + η*_t_*, where

$$f_{\text{env}}\left(s_{t},a_{t}\right)=\left[\begin{array}{c} s_{x_{t}}+\Delta t\;s_{v_{t}}\text{cos}s_{\theta_{t}}\\ s_{y_{t}}+\Delta t\;s_{v_{t}}\text{sin}s_{\theta_{t}}\\ s_{\theta_{t}}+\Delta t\;s_{\omega_{t}}\\ n\;G_{v}a_{v_{t}}+p_{v_{t}}\\ n\;G_{\omega }a_{\omega_{t}}+p_{\omega_{t}}\\ g_{x_{t}}\\ g_{y_{t}} \end{array}\right]$$ (1)

and zero-mean independent Gaussian process noise added to the velocities

$$\eta_{t}={\left[0,0,0,\eta_{v_{t}},\eta_{\omega_{t}},0,0\right]}^{⊤},\;{\left[\eta_{v_{t}},\eta_{\omega_{t}}\right]}^{⊤}∼N\left[0,\text{diag}\left(\sigma_{a}^{2}\right)\right]$$

with SD σ*_a_* = [σ*_a_v__*, σ_*a*_ω__]^⊤^. The operator diag(·) constructs a diagonal matrix with its vector argument on the diagonal. The time step is Δ*t* = 0.1 s. Joystick gain **G** = [*G_v_*, *G*_ω_]^⊤^ = [200 cm/s, 90°/s]^⊤^ maps dimensionless linear and angular joystick actions to units of velocities. Gain multiplier *n* scales **G**. Linear and angular perturbation velocities are *p_v_t__* and *p*_ω*_t_*_.

#### Reward

The reward function *R*(**s***_t_*, **a***_t_*) maps a state-action pair to a scalar *r_t_*. We firstly defined an action threshold a^*^ = 0.1 to distinguish between when the agent had not yet begun moving and when they moved and stopped: The agent must increase the magnitude of at least one action component above a^*^ in the beginning (start criterion), and then the agent must reduce the magnitude of both action components below a^*^ to indicate a stop (stop criterion). Nonzero rewards were only offered in the last step of each trial and if the agent satisfied both criteria. For the nonzero rewards, we defined **d***_t_* = [*s_x_t__*, *s_y_t__*]^⊤^ − [*g_x_t__*, *g_y_t__*]^⊤^ as the displacement between the agent’s and the target’s locations, and a reward *r_t_* = 10 would be given if the Euclidean distance ∥**d***_t_*∥_2_ was smaller than the radius of the reward zone d^*^ = 65 cm. To facilitate training in the early stages, we allowed a small reward $r_{t}=10\text{exp}\left(-0.5d_{t}^{⊤}\Sigma_{r}^{-1}d_{t}\right)$ if the agent stopped outside the reward zone, where Σ*_r_* = (d^*^/1.5)^2^*I*_2_ is a constant matrix, and *I*_2_ denotes the identity matrix of size 2.

#### Done

A trial ended when the agent stopped, or if *t* exceeded the maximum trial duration 3.4 s. For later convenience, let *D_t_* denote a trial completion flag that equals 1 if the trial is done at *t* otherwise 0. A new trial thereafter started with a new sampled initial state **s**_0_.

#### Observation

**o***_t_* ∈ Ω is a vector [*o_v_t__*, *o*_ω*_t_*_, *o*_*g*_*x*,*t*__, *o*_*g*_*y*,*t*__]^⊤^ containing observations of the agent’s linear and angular velocities through optic flow and the target’s *x* and *y* positions when visible in the first 0.3 s of each trial. **o***_t_* ∼ *O*(**o***_t_* ∣ **s***_t_*) was defined as

$$o_{t}=H_{t}s_{t}+\zeta_{t}$$ (2)

where ζ*_t_* is a zero-mean Gaussian observation noise, and the observation model *H_t_* is a 4 × 7 matrix filled mostly with zeros, except for a few observable elements depending on the time within a trial: When *t* ≤ 0.3 s, the target is visible, so *H*^1,4^, *H*^2,5^, *H*^3,6^, and *H*^4,7^ are equal to 1, where superscripts denote row and column; after *t* = 0.3 s, the target disappears and only the optic flow is observable, so only *H*^1,4^ and *H*^2,5^ are 1. For the observation noise, ζ_0_ = 0, ζ_*t*>0_ = [ζ*_v_t__*, ζ*_ω_t__*,0,0]^⊤^, where ζ*_v_t__* and ζ_ω*_t_*_ denote linear and angular observation noises, and ${\left[\zeta_{v_{t}},\zeta_{\omega_{t}}\right]}^{⊤}∼N\left[0,\text{diag}\left(\sigma_{o}^{2}\right)\right]$ with SD σ*_o_* = [σ*_o_v__*, σ_*o*_ω__]^⊤^.

### Task parameters

#### Training task

The gain multiplier in Eq. 1 is given by *n* = 1. There were no perturbations, so for any *t*, *p_v_t__* = *p*_ω_*t*__ = 0. Process and observation noise SDs were in units of **G**, i.e., σ*_a_* = α*_a_***G** and σ*_o_* = α*_o_***G**. α*_a_*, α*_o_* ∈ {0,0.1,0.2,0.3} were used in Fig. 7. α*_a_* = 0.4 and α*_o_* = 0.1 were used in fig. S2 (C and D). α*_a_* = 0.2 and α*_o_* = 0.1 were used to train agents for all other figures.

#### Gain task

The gain multiplier *n* was increased to values greater than 1 for *n*× gain. The gain for each analysis was specified in its caption. Noise SDs were also multiplied by the same gains, i.e., **σ***_a_* = α*_a_n***G**, **σ***_o_* = α*_o_n***G**. There were no perturbations.

#### Perturbation task

Parameters *n*, σ*_a_*, and σ*_o_* were the same as those in the training task. There were three perturbation parameters sampled for each trial: perturbation peak time relative to the trial start *t_p_* and perturbation linear and angular peaks *p*_*v*_peak__ and *p*_*w*_peak__. These parameters were sampled from the ranges specified in the caption for each analysis. The sampled perturbation parameters determined Gaussian-shaped linear and angular perturbations in Eq. 1, defined as

$${\left[p_{v_{t}},p_{\omega_{t}}\right]}^{⊤}=\left\{\begin{array}{cc} {\left[p_{v_{\text{peak}}},p_{\omega_{\text{peak}}}\right]}^{⊤}\cdot \text{exp}\left[-\frac{1}{2}{\left(\frac{t-t_{p}}{0.2}\right)}^{2}\right], & \text{if}\;t_{p}-0.5\leq t\leq t_{p}+0.5\\ {\left[0,0\right]}^{⊤}, & \text{otherwise} \end{array}\right.$$

### Belief modeling

The state **s***_t_* is partially observable in our task; therefore, an agent cannot make a good decision only based on the current sensory inputs. Instead, it benefits from maintaining a belief state representation *b_t_*, which is a posterior of **s***_t_*, for decision-making. We considered both a model-based inference method and a gradient-based optimization method to model the belief.

#### Recursive Bayesian estimation

When the transition probability *T* and the observation probability *O* are known, the belief is a posterior of **s***_t_* given all available observations and actions, i.e., *b_t_* = *p*(**s***_t_* ∣ **o**_0:*t*_, **a**_0:*t*−1_), and can be inferred recursively as

$$b_{t}=\frac{1}{C}O\left(o_{t}|s_{t}\right)\int_{s_{t-1}}T\left(s_{t}|s_{t-1},a_{t-1}\right)\;b_{t-1}\;ds_{t-1}$$ (3)

where *C* = *p*(**o***_t_* ∣ **o**_0:*t*−1_, **a**_0:*t*−1_) is a normalization constant, and *b*_*t*−1_ = *p*(**s**_*t*−1_ ∣ **o**_0:*t*−1_, **a**_0:*t*−2_).

#### EKF belief

When all variables are Gaussian in the recursive Bayesian estimation and *T* is nonlinear, the EKF (*31*) is a tractable method that uses a local linearization to approximate Eq. 3. The belief here is a Gaussian density $b_{t}=N\left({\hat{s}}_{t},P_{t}\right)$ . To simplify the computation here, we express position in relative coordinates by letting the initial belief mean be ${\hat{s}}_{0}={\left[{\hat{s}}_{x_{0}},{\hat{s}}_{y_{0}},{\hat{s}}_{\theta_{0}},{\hat{s}}_{v_{0}},{\hat{s}}_{\omega_{0}}\right]}^{⊤}={\left[-g_{x_{0}},-g_{y_{0}},90,0,0\right]}^{⊤}$ and let the state transition **f**_env_ contain the first five equations in Eq. 1 to reduce the dimensionality of the state by two. Let ϵ denote a small number 10^−8^, we defined the initial belief covariance *P*_0_ = ϵ*I*_5_. Let a 5 × 5 matrix Σ*_a_* denote the Gaussian process noise covariance filled with 0 except $\Sigma_{a}^{4,4}=\sigma_{a_{v}}^{2}$ , $\Sigma_{a}^{5,5}=\sigma_{a_{\omega }}^{2}$ . The observation’s dimensionality was reduced by two by omitting the target location, yielding **o***_t_* = [*o_v_t__*, *o*_ω*_t_*_]^⊤^. The observation model *H* in Eq. 2 then becomes a 2 × 5 matrix filled with 0, except *H*^1,4^ = *H*^2,5^ = 1. Let $\Sigma_{o}=\text{diag}\left(\sigma_{o}^{2}\right)$ denote the Gaussian observation noise covariance. Any 0 variance components were replaced with a minimal variance of ϵ for Σ*_a_*, Σ*_o_*.

**f**_env_ at $b_{t-1}=N\left({\hat{s}}_{t-1},P_{t-1}\right)$ was locally linearized as

$$A_{t-1}=\frac{\partial f_{\text{env}}}{\partial {\hat{s}}_{t-1}}=\left[\begin{array}{ccccc} 1 & 0 & -{\hat{s}}_{v_{t-1}}\Delta t\text{sin}{\hat{s}}_{\theta_{t-1}} & \Delta t\text{cos}{\hat{s}}_{\theta_{t-1}} & 0\\ 0 & 1 & {\hat{s}}_{v_{t-1}}\Delta t\text{cos}{\hat{s}}_{\theta_{t-1}} & \Delta t\text{sin}{\hat{s}}_{\theta_{t-1}} & 0\\ 0 & 0 & 1 & 0 & \Delta t\\ 0 & 0 & 0 & 0 & 0\\ 0 & 0 & 0 & 0 & 0 \end{array}\right]$$

The EKF’s prediction step (Eq. 4) uses **a**_*t*−1_ to get a predicted belief *b*_*t*∣*t*−1_. Note that given our *A*_*t*−1_, velocity variance elements in the prediction $P_{t|t-1}^{4,4},P_{t|t-1}^{5,5}$ only depend on Σ*_a_* and independent of *P*_*t*−1_.

$$\begin{array}{c} {\hat{s}}_{t|t-1}=f_{\text{env}}\left({\hat{s}}_{t-1},a_{t-1}\right)\\ P_{t|t-1}=A_{t-1}P_{t-1}A_{t-1}^{⊤}+\Sigma_{a} \end{array}$$ (4)

The EKF’s update step (Eq. 5) uses *b*_*t*∣*t*−1_ and **o***_t_* to get the final belief $b_{t}=N\left({\hat{s}}_{t},P_{t}\right)$ . *K_t_* is known as the Kalman gain which specifies the relative weights of prediction and observation. Mathematically, because only velocity components are observable and predicted velocity components are independent of *P*_*t*−1_, *K_t_* is determined solely by Σ*_a_* and Σ*_o_* and is independent of *P*_*t*−1_ in our task

$$\begin{array}{c} K_{t}=P_{t|t-1}H^{⊤}{\left(HP_{t|t-1}H^{⊤}+\Sigma_{o}\right)}^{-1}\\ {\hat{s}}_{t}={\hat{s}}_{t|t-1}+K_{t}\left(o_{t}-H{\hat{s}}_{t|t-1}\right)\\ P_{t}=\left(I_{5}-K_{t}H\right)P_{t|t-1} \end{array}$$ (5)

#### RNN belief

When the transition and the observation probabilities *T*, *O* are unknown to the agent, to support decision-making, an internal belief could be formed via gradient-based optimization. We used RNNs to integrate partial observations **o***_t_* and motor efference copies **a**_*t*−1_ over time, trained end-to-end using the RL objective in our task (see below). RNN’s belief *b_t_* resides in its hidden state **h***_t_*. Each RNN maintains a hidden state **h***_t_* = **f**_RNN_(**o***_t_*, **a**_*t*−1_, **h**_*t*−1_) or **h***_t_* = **f**_RNN_(**o***_t_*, **a**_*t*−1_, **h**_*t*−1_, **a***_t_*) depending on its inputs (Fig. 8, A and B). *b_t_* encoded implicitly in **h***_t_* is used by other neurons to compute **a***_t_* or *Q_t_* in the actor or critic.

### RL with EKF beliefs

Our RL algorithm for training the EKF agent with an EKF belief (used for Fig. 2B) is based on an actor-critic approach called the twin delayed deep deterministic policy gradient (TD3) (*29*), referred to as EKF-TD3. We first computed beliefs using EKF as described above and then trained neural networks to use those beliefs as inputs to guide actions.

#### Networks

Each agent has two critics with identical architectures but different initial weights to address the maximization bias in value estimation [see the “Critic update” section below and (*51*)], although in the Results we only showed one of the critics used to train the actor to generate actions. Let **i***_t_* denote the state-related inputs. All neural networks in an EKF agent were feed-forward, provided with the mean and covariance of *b_t_* computed by the EKF, i.e., $i_{t}=\left\{{\hat{s}}_{t},P_{t}\right\}$ . The actor and two critics are ${\hat{a}}_{t}=\pi_{\mu }\left(i_{t}\right)$ , *Q*_*t*_1__ = 𝒬_ν_1__(**i***_t_*, **a***_t_*), and *Q*_*t*_2__ = 𝒬_ν_2__(**i***_t_*, **a***_t_*), where μ, ν_1_, and ν_2_ denote neural parameters.

#### Exploration

Since our actor is a deterministic function, to realize exploration in training, we combined the actor’s output ${\hat{a}}_{t}=\pi_{\mu }\left(i_{t}\right)$ with a zero-mean Gaussian exploration noise β*_t_* and clipped the sum to the box [−1,1]

$$a_{t}=\text{clip}\left({\hat{a}}_{t}+\beta_{t},-1,1\right),\;\;\beta_{t}∼N\left(0,\sigma_{\text{exp}}^{2}I_{2}\right)$$ (6)

To ensure that the agent can properly stop without noise variability, we let β*_t_* = 0 if ${\hat{a}}_{t}$ is below the action threshold. After training, we also let β*_t_* = 0, so $a_{t}={\hat{a}}_{t}$.

#### Experience replay

Instead of learning on the current trial, we used off-policy RL by storing experience in a replay buffer *ℬ* and frequently sampling data from *ℬ* to train the agent. At each state **s***_t_*, the EKF computed **i***_t_* for the actor to generate **a***_t_* following Eq. 6. The agent observed the reward *r_t_*, next input **i**_*t*+1_, and trial completion flag *D_t_* and stored the one-step transition tuple (**i***_t_*, **a***_t_*, *r_t_*, **i**_*t*+1_, *D_t_*) in *ℬ*. The buffer *ℬ* had a capacity of 1.6 × 10^6^ transitions, storing data on a first-in, first-out (FIFO) basis. Furthermore, we augmented the experience by also storing the mirror transition $\left({\hat{ı}}_{t},{\hat{a}}_{t},r_{t},{\hat{ı}}_{t+1},D_{t}\right)$ generated by reflecting the original data across the *y* axis.

#### Target networks

The learning of value in TD3 is akin to deep Q-learning (*34*). Using the Bellman equation, ideally, the agent can learn to estimate the value 𝒬*_ν_j__*(**i***_t_*, **a***_t_*) by regressing the learning target *y_t_* = *r_t_* + γ𝒬*_ν_j__*[**i**_*t*+1_, π_μ_(**i**_*t*+1_)], i.e., the one-step bootstrapping of the value after receiving the reward *r_t_*, observing the next input **i**_*t*+1_, and estimating the next action π_μ_(**i**_*t*+1_). One stability issue here is that the neural parameters for optimization are also used to construct the learning target *y_t_*, which changes at each learning step. To obtain a more stable *y_t_*, we thus maintained a copy of actor and critic networks with more slowly changing parameters μ′ and ν*_j_*′ used in *y_t_*, referred to as target actor and critic networks. These parameters were initialized to be the same as μ, ν*_j_* and passed through an exponential moving average

$$\begin{array}{c} \mu \prime \leftarrow \tau \mu +\left(1-\tau \right)\mu \prime \\ \nu \prime_{j}\leftarrow \tau \nu_{j}+\left(1-\tau \right)\nu \prime_{j} \end{array}$$ (7)

We used τ = 0.005.

#### Critic update

We sampled a batch of *M* = 256 transitions from the buffer each time

$${\left[i^{\left(k\right)},a^{\left(k\right)},r^{\left(k\right)},i^{\prime (k)},D^{\left(k\right)}\right]}_{k=1,2,\cdots ,M}∼ℬ$$

where the temporal subscript is omitted, and **i**′^(^*^k^*^)^ denotes the next input after **i**^(*k*)^. The next action given **i**′^(^*^k^*^)^ was estimated by the target actor network as

$$a^{\prime (k)}=\text{clip}\left\{\pi_{\mu \prime }\left[i^{\prime (k)}\right]+\beta^{\prime (k)},-1,1\right\},\;\;\beta^{\prime (k)}∼\text{clip}\left[N\left(0,0.05^{2}I_{2}\right),-0.1,0.1\right\}$$ (8)

where β′^(*k*)^ is small zero-mean Gaussian noise clipped to [−0.1,0.1] to smooth the action estimation.

The learning target *y*^(*k*)^ used the smaller value estimation between two target critics to reduce the maximization bias (*51*) and was truncated at the end of each trial [*D*^(*k*)^ = 1]. The learning objective of the two critics, *J*(ν*_j_*), *j* = 1,2, was to regress the learning target *y*^(*k*)^, defined as

$$\begin{array}{c} y^{\left(k\right)}=r^{\left(k\right)}+\left[1-D^{\left(k\right)}\right]\;\gamma \underset{j=1,2}{\text{min}}Q_{\nu_{j}\prime }\left[i^{\prime (k)},a^{\prime (k)}\right]\\ J\left(\nu_{j}\right)=\frac{1}{M}\sum_{k=1}^{M}{\left\{y^{\left(k\right)}-Q_{\nu_{j}}\left[i^{\left(k\right)},a^{\left(k\right)}\right]\right\}}^{2} \end{array}$$ (9)

The gradient ∇*_ν_j__J*(*ν_j_*) was computed by BP. Critic parameters ν*_j_* were updated (see the “Agent training” section below for optimizers) using ∇*_ν_j__J*(*ν_j_*) to minimize *J*(*ν_j_*).

#### Actor update

The actor’s parameter μ was updated once for every two critic updates. The actor’s learning objective *J*(μ) was to maximize the value of the first critic, defined as

$$J\left(\mu \right)=\frac{1}{M}\sum_{k=1}^{M}Q_{\nu_{1}}\left\{i^{\left(k\right)},\pi_{\mu }\left[i^{\left(k\right)}\right]\right\}$$ (10)

The gradient ∇_μ_*J*(μ) was computed by BP. The actor parameter μ was updated using ∇_μ_*J*(μ) to maximize *J*(μ). Note that the critic parameter ν_1_ was not updated here. A diagram illustrating the critic and actor update is shown in fig. S1 (A and B).

### RL with RNN beliefs

We developed a memory-based TD3 model leveraging RNNs to construct a form of internal beliefs to tackle POMDPs, referred to as RNN-TD3. All agents except the EKF agent were trained by this algorithm.

#### Networks

Let **i***_t_* = {**o***_t_*, **a**_*t*−1_} and **h***_t_* denote the state-related inputs and the RNN’s hidden state. The actor and two critics are $\left\{{\hat{a}}_{t},h_{t}^{\mu }\right\}=\pi_{\mu }\left(i_{t},h_{t-1}^{\mu }\right)$ , $\left\{Q_{t_{j}},h_{t}^{\nu_{j}}\right\}=Q_{\nu_{j}}\left(i_{t},a_{t},h_{t-1}^{\nu_{j}}\right),j=1,2$ , where networks’ beliefs are implicitly encoded in all **h***_t_* evolving over time. At the beginning of each trial, **h**_*t*−1_ and **a**_*t*−1_ were initialized to zeros. For simplicity, we drop **h***_t_* in our notations for all networks’ outputs.

#### Exploration

Similar to that of EKF-TD3 (Eq. 6), we added zero-mean Gaussian exploration noise to the output of the actor ${\hat{a}}_{t}=\pi_{\mu }\left(i_{t},h_{t-1}^{\mu }\right)$ if the output is above the action threshold

$$a_{t}=\text{clip}\left({\hat{a}}_{t}+\beta_{t},-1,1\right),\;\;\beta_{t}∼N\;\left(0,\sigma_{\text{exp}}^{2}I_{2}\right)$$ (11)

After training, we let β*_t_* = 0.

#### Experience replay

Similar to that of EKF-TD3 but rather than storing one-step transition tuples, the replay buffer *ℬ* stored the whole trajectory for each trial of *N* time steps

$$\left(i_{0},a_{0},r_{0},D_{0},\cdots ,i_{N-1},a_{N-1},r_{N-1},D_{N-1}\right)$$

and its mirror image, because RNNs have hidden states **h***_t_* generally depend on the entire history of inputs, not just the most recent ones. Each action was obtained using Eq. 11. The FIFO buffer had a capacity of 10^5^ trajectories.

#### Target networks

Same as that of EKF-TD3.

#### Critic update

Similar to that of EKF-TD3, but critics here also needed to learn the temporal structure. Since the trial duration *N* varies across trials, we first sampled a trial duration $\tilde{N}$ from the buffer *ℬ* and then sampled a batch of *M* = 16 trajectories with the same duration $\tilde{N}$

$${\left[i_{t}^{\left(k\right)},a_{t}^{\left(k\right)},r_{t}^{\left(k\right)},i_{t}^{\prime (k)},D_{t}^{\left(k\right)}\right]}_{t=0,\cdots ,\tilde{N}-1}^{k=1,\cdots ,M}∼ℬ$$

where $i_{t}^{\prime (k)}=i_{t+1}^{\left(k\right)}$ . The next action $a_{t}^{\prime (k)}$ , the learning target $y_{t}^{\left(k\right)}$ , and the learning objective of the two critics *J*(ν*_j_*) were

$$a_{t}^{\prime (k)}=\text{clip}\left\{\pi_{\mu \prime }\left[i_{t}^{\prime (k)},h_{t}^{\mu^{\prime (k)}}\right]+\beta_{t}^{\prime (k)},-1,1\right\},\;\;\beta_{t}^{\prime (k)}∼\text{clip}\left[N\left(0,0.05^{2}I_{2}\right),-0.1,0.1\right]$$ (12)

$$\begin{array}{c} y_{t}^{\left(k\right)}=r_{t}^{\left(k\right)}+\left[1-D_{t}^{\left(k\right)}\right]\;\gamma \underset{j=1,2}{\text{min}}Q_{\nu_{j^{\prime }}}\left[i_{t}^{\prime (k)},a_{t}^{\prime (k)},h_{t}^{{\nu_{j}}^{\prime (k)}}\right]\\ J\left(\nu_{j}\right)=\frac{1}{M\tilde{N}}\sum_{k=1}^{M}\sum_{t=0}^{\tilde{N}-1}{\left\{y_{t}^{\left(k\right)}-Q_{\nu_{j}}\left[i_{t}^{\left(k\right)},a_{t}^{\left(k\right)},h_{t-1}^{\nu_{j}\left(k\right)}\right]\right\}}^{2} \end{array}$$ (13)

The gradient ∇*_ν_j__J*(*ν_j_*) was computed by BP through time (BPTT). Critic parameters ν*_j_* were updated using ∇*_ν_j__J*(ν*_j_*) to minimize *J*(ν*_j_*).

#### Actor update

Similar to that of EKF-TD3, but the actor here needed to learn the temporal structure. The actor’s learning objective *J*(μ) was

$$J\left(\mu \right)=\frac{1}{M\tilde{N}}\sum_{k=1}^{M}\sum_{t=0}^{\tilde{N}-1}\;Q_{\nu_{1}}\left\{i_{t}^{\left(k\right)},\pi_{\mu }\left[i_{t}^{\left(k\right)},h_{t-1}^{\mu \left(k\right)}\right],h_{t-1}^{\nu_{1}\left(k\right)}\right\}$$ (14)

The gradient ∇_μ_*J*(μ) was computed by BPTT. The actor parameters μ were updated using ∇_μ_*J*(μ) to maximize *J*(μ).

### Agent training

All network parameters μ, ν_1_, and ν_2_ were updated by the Adam optimizers (*52*). Optimizer parameters were set as follows: learning rates annealed from 3 × 10^−4^ to 5 × 10^−5^, exponential decay rates for the first and second moment estimates =0.9,0.999, a constant added in denominators for numerical stability =1.5 × 10^−4^, and weight decay =0. The critics were updated once for every *c* = 4 interactions with the environment. The actor was updated once for every two critic updates.

During training, we periodically validated the agent’s performance with 300 validation trials and used the moments when the agent achieved 20 and 80% accuracy to split the whole training course into three phases. The learning rates for the actor and critics, the exploration noise σ_exp_ (Eqs. 6 and 11), and the observation noise σ*_o_* (Eq. 2) in each phase were set as follows: In phase 1, learning rates were 3 × 10^−4^, σ_exp_ = 0.8, and σ*_o_* = 0. In phase 2, learning rates were 3 × 10^−4^, σ_exp_ = 0.5, and σ*_o_* = α*_o_***G**, where α*_o_* is defined in the training task. In phase 3, learning rates were 5 × 10^−5^, σ_exp_ = 0.4, and σ*_o_* = α*_o_***G**. Training was stopped after the agent had experienced 10^5^ trials after phase 1 (extensive training) for Fig. 8 and figs. S2 (E and F) and S7 to S9 or 10^4^ trials after phase 1 (default training) for the remaining agents. We summarize the EKF/RNN-TD3 algorithms in algorithm S1 and hyperparameters in table S1.

### Agent testing and TD errors

During testing, the target networks were no longer used. The trained actor π_μ_ was used to interact with the environment and generate transition tuples $\left(i_{t},a_{t},r_{t},i\prime_{t},D_{t}\right)$ for each *t*. No exploration noise was added to the output of the actor.

The TD error shown in analyses is similar to the learning objective for critics (Eq. 13), except that the target networks were replaced with the trained networks, and only the first critic was used. Specifically, for each *t*, the next action is $a\prime_{t}=\pi_{\mu }\left(i\prime_{t},h_{t}^{\mu }\right)$, and the TD error is given by

$$|r_{t}+\left(1-D_{t}\right)\;\gamma Q_{\nu_{1}}\left(i\prime_{t},a\prime_{t},h_{t}^{\nu_{1}}\right)-Q_{\nu_{1}}\left(i_{t},a_{t},h_{t-1}^{\nu_{1}}\right)|$$

### Agent selection

After training phase 1, in every 500 training trials, we saved neural parameters of each network. To fairly compare agents’ performance in each task (training, gain, and perturbation), we tested all sets of stored parameters (without exploration noise) for each task using one or multiple test sets, with 500 trials (for agents that underwent default training) or 300 trials (for agents that underwent extensive training) each. We then endowed each agent with the neural parameters that allowed it to achieve the highest reward rate averaged across test sets for each task.

The test sets used for each task are as follows. Training task: One test set with the training task’s parameters. Gain task: Three or four test sets with the gain = 1×,1.5×,2× (default training) or 1×,2×,3×,4× (extensive training). Perturbation task: Two test sets for agents that underwent default training, one without perturbation and the other with perturbation parameters identical to those in Fig. 6C. For agents that underwent extensive training, an additional test set was included, with perturbation ranges identical to those in Fig. 8D.

### Agent architectures

Although all agents had two architecturally identical critics, we only showed one in Figs. 1E and 8A. All RNNs were implemented as long short-term memory (LSTM) networks (*53*), as we observed that agents with LSTMs were much easier to train than those with vanilla RNNs. Agents with vanilla RNNs encountered learning failures most of the time. In contrast to LSTMs, the training of vanilla RNNs proves to be unstable, primarily because they struggle with managing long-term dependencies and are susceptible to vanishing/exploding gradient problems (*53*). These inherent difficulties may be exacerbated with RL, as the learning target for the critic network is bootstrapped by the critic itself (see above). Consequently, critic updates are preferable to be stable to ensure a more stable learning target. Previous work using RNNs within critics consistently chose RNNs with gating mechanisms (*39*, *54*–*56*). It remains a prospect for future research to investigate the impact of the gating inductive bias in RL.

All MLP layers linearly transformed inputs and then applied rectified linear unit (ReLU) nonlinearities. The output of critics *Q_t_* was produced by a linear unit without any nonlinearity; the linear and angular control outputs of the actors **a***_t_* were bounded to [−1,1] by hyperbolic tangent nonlinearities. In the holistic critic/actor (Fig. 8, A and B), there were 220 LSTM units. In all other architectures in Fig. 8 (A and B), each RNN module had 128 LSTM units, and each MLP module contained two layers with 300 ReLU units in each. All architectures, as a result, had a similar number of parameters (fig. S9A).

The EKF agent’s actor and two critics used the same architecture consisting of an MLP module with two layers, each with 300 ReLU units. The holistic EKF agent used an actor architecture identical to the EKF agent’s actor and a holistic critic architecture.

### No generalization hypothesis

For each gain trial with a gain *n***G** for *n* > 1 or for each perturbation trial with nonzero perturbation velocities *p_v_t__*, *p_w_t__*, the hypothetical no generalization trajectory was obtained as follows. We first recorded the agent/monkey’s sequential actions (**a**_0_, **a**_1_, …, **a**_*N*−1_) in the training task (1× gain, no perturbations) navigating to the same target (for agents) or the closest target in the dataset (for monkeys). We then regenerated a new trajectory using (**a**_0_, **a**_1_, …, **a**_*N*−1_) following the environmental transition (Eq. 1, process noise η*_t_* = 0), but with the gain multiplier *n* for the gain task or the perturbation velocities *p_v_t__*, *p_w_t__* for the perturbation task.

### Under/overshooting definition using idealized circular trajectories

To determine when an agent or a monkey under- or overshot the target in the gain task, we asked whether its stop location exceeded the target location in the distance along their corresponding idealized circular trajectories. Specifically, given an arbitrary endpoint ${\left[\tilde{x},\tilde{y}\right]}^{⊤}$ , the circular trajectory connecting it from a forward heading (90°, initial head direction) at the origin (start location) has a radius as a function of this point $\tilde{R}\left(\tilde{x},\tilde{y}\right)$ . The arc length of this trajectory is a function

$$L\left(\tilde{x},\tilde{y}\right)=2\tilde{r}\text{arcsin}\left(\frac{\sqrt{{\tilde{x}}^{2}+{\tilde{y}}^{2}}}{2\tilde{r}}\right),\;\tilde{r}=\tilde{R}\left(\tilde{x},\tilde{y}\right)=\frac{{\tilde{x}}^{2}+{\tilde{y}}^{2}}{2\tilde{x}}$$

We deemed the agent’s stop location [*s*_*x*_*N*−1__, *s*_*y*_*N*−1__]^⊤^ to have overshot the target [*g_x_*, *g_y_*]^⊤^ if *L*(*s*_*x*_*N*−1__, *s*_*y*_*N*−1__) > *L*(*g_x_*, *g_y_*), otherwise it undershot.

### Trajectory length and curvature

We approximated the length $\tilde{l}$ and the curvature ${\tilde{k}}_{t}$ of a trajectory (*s_x_t__*, *s_y_t__*)_*t*=0,⋯,*N*−1_ as follows

$$\begin{array}{c} \tilde{l}=\sum_{t=0}^{N-2}\sqrt{{\left(s_{x_{t+1}}-s_{x_{t}}\right)}^{2}+{\left(s_{y_{t+1}}-s_{y_{t}}\right)}^{2}}\\ {\tilde{k}}_{t}=\frac{|{s_{x_{t}}}^{\prime }{s_{y_{t}}}^{\prime \prime }-{s_{y_{t}}}^{\prime }{s_{x_{t}}}^{\prime \prime }|}{{\left({s_{x_{t}}}^{\prime 2}+{s_{y_{t}}}^{\prime 2}\right)}^{\frac{3}{2}}} \end{array}$$

where first derivatives $s_{x_{t}}\prime ,s_{y_{t}}\prime$ and second derivatives ${s_{x_{t}}}^{\prime \prime },{s_{y_{t}}}^{\prime \prime }$ were estimated using first-order one-sided differences for the first and last points and second-order central differences for interior points. In each trial, we excluded curvatures that surpassed the 95th percentile at any step, considering them as outlier values. Note that the monkeys’ trajectories here were downsampled to have the same 0.1-s time step as the agents’ trajectories.

### Spatial tuning

We obtained the approximate spatial tuning of each neuron by linearly interpolating its activity and the agent’s *x* and *y* location using data from each step across trials, followed by a convolution over the 2D space using a boxcar filter with a height and a width of 40 cm.

### Neural decoding

While agents were being tested, we recorded their sensory, latent, and motor variables for the analyses in Fig. 1L and fig. S1F and their positions *s_x_t__*, *s_y_t__* for all other decoding analyses. We also recorded their neural activities in each module for both their actors and critics. Let *S* denote a partitioned matrix where rows are time steps and columns are decoding target variables, e.g., [**s***_x_*, **s***_y_*] for agent’s positions. Recorded neural activities *X* were concatenated over time, where rows are time steps and columns are units. A linear decoder regressed *S* on *X*, whose partitioned parameters for all decoding variables *W* were obtained by the ridge estimator following

$$W={\left(X^{⊤}X+\lambda I\right)}^{-1}X^{⊤}S$$

where λ is a penalty term chosen from {0.1,1,10} by cross-validation. We always used 70% trials in the dataset to train the decoder and used the remaining 30% trials to test the decoder’s predictions.

The decoding error of the belief in each trial was defined as

$$\frac{1}{N}\sum_{t=0}^{N-1}∥{\hat{s}}_{x_{t}}-s_{x_{t}},{\hat{s}}_{y_{t}}-s_{y_{t}}{∥}_{2}$$

where ${\hat{s}}_{x_{t}},{\hat{s}}_{y_{t}}$ are predicted *x* and *y* positions.

### Statistical analysis

All agents were trained with eight different random seeds, which determined the initialized neural network parameters and random variables in training (e.g., process and observation noises, agent’s initial state, exploration noise, and sampling from the buffer). All analyses for agents included data from training runs with all random seeds unless otherwise noted. We reported mean, SD, SEM, or confidence interval (CI) throughout the paper. All correlations were quantified by Pearson’s *r*.

In all violin plots, we determined upper and lower whiskers following *q*_1_ − whis · (*q*_3_ − *q*_1_) and *q*_1_ + whis · (*q*_3_ − *q*_1_), where *q*_1_ and *q*_3_ are the first and third quartiles, and whis = 1.5 (*57*). We did not plot outliers beyond the whisker range for better visualization, but we did not exclude them in quantification.

## Supplementary Materials

### This PDF file includes:

Supplementary Text Figs. S1 to S9 Table S1 Algorithm S1 References
