# Supplementary material for: Inductive biases of neural network modularity in spatial navigation
Source: Sci Adv. 2024 Jul 19;10(29):eadk1256. doi: 10.1126/sciadv.adk1256 (PMC11259174; doi:10.1126/sciadv.adk1256)
Supplement: Supplementary file 1 — Supplementary Text Figs. S1 to S9 Table S1 Algorithm S1 References [file sciadv.adk1256_sm.pdf]

Science Advances
AAAS

Supplementary Materials for

Inductive biases of neural network modularity in spatial navigation

Ruiyi Zhang, *et al.*

Corresponding author: Ruiyi Zhang, rz31@nyu.edu

Sci. Adv. **10**, eadk1256 (2024)
DOI: 10.1126/sciadv.adk1256

This PDF file includes:

Supplementary Text
Figs. S1 to S9
Table S1
Algorithm S1
References

Supplementary Text

The difference between animal and agent learning

Our agent learning algorithm uses off-policy RL with biologically implausible mechanisms to enhance training. For instance, agents learn from sampled trajectories stored in a large replay buffer and use additional auxiliary networks (29). The update of neural parameters involves back-propagation through time, as opposed to more biologically-inspired algorithms. Details on agent training are provided in Materials and Methods.

Agents also learn this task from scratch, unlike animals that may possess pre-existing knowledge derived from other experience, such as an understanding of the physics of movement. Agents lack such prior knowledge, and in the initial stages of training they often experience long periods of stagnation, relying solely on random exploration to receive the first few rewards (58). Additionally, animals can partially or fully learn new tasks based on their specific needs. The generalization behaviors in our specific tasks show similarities between animals and agents, as animals also demonstrated zero-shot generalization and showed no further improvement when tested on novel tasks (25). However, it is crucial not to assume the universality of this across all tasks. For instance, animals can update their internal models for prediction when faced with novel state transitions (50)—a capability absent in our agents, which must consistently rely on observation.

We induced agents to rely more on observation by introducing higher prediction uncertainty than observation uncertainty during training. Monkeys in our task also exhibited a preference for observation over prediction (26). However, the origin of this preference in animals remains unclear. One possibility is that, similar to agents, monkeys learned this preference from the task itself. Alternatively, as visual animals, this preference may be inherent in the monkey brain before learning of our task. Future experiments could be designed to better control observation and prediction uncertainties, allowing for a more in-depth investigation into the 'Kalman gain' of monkeys.

Learning an EKF-like belief in RNNs

Typically, an EKF computation is decomposed into two steps—prediction and update. The update step weighs the result from the prediction step and observation. An RNN, as a universal function approximator, should learn to approximate an EKF computation with proper training. However, following the idea of modularization, if we consider decomposing the approximation of an EKF into the prediction and update steps using two neural modules, this may serve as a useful inductive bias.

However, for our specific task, the two-step EKF computation can be easily consolidated into a single step (also see Materials and Methods); therefore, it is not an appropriate task to test the advantage of using two neural modules, each in charge of an EKF step.

To clarify, let us walk through the EKF computation for our task. the belief b_t comprises a positional component b_t^p and a velocity component b_t^v, i.e., $b_t = \{b_t^p, b_t^v\}$. In the prediction step from $t-1$ to t, b_t^p is predicted through path integration only using the last belief b_{t-1}, i.e., $b_{t|t-1}^p = \boldsymbol{f}_1(b_{t-1}^p, b_{t-1}^v) = \boldsymbol{f}_1(b_{t-1})$. Simultaneously, b_t^v is predicted based solely on the last action $\boldsymbol{a}_{t-1}$ (joystick gain times $\boldsymbol{a}_{t-1}$), i.e., $b_{t|t-1}^v = \boldsymbol{f}_2(\boldsymbol{a}_{t-1})$. Moving on to the update step, the positional belief remains unaltered by the observation, as the position information is unobservable, i.e., $b_t^p = b_{t|t-1}^p$. However, the velocity belief weighs the predicted and observed velocities according to the Kalman gain, expressed as $b_t^v = \boldsymbol{f}_3(b_{t|t-1}^v, \boldsymbol{o}_t)$. Importantly, since $b_{t|t-1}^v$ is directly proportional to $\boldsymbol{a}_{t-1}$, b_t^v essentially weighs $\boldsymbol{a}_{t-1}$ and $\boldsymbol{o}_t$, i.e., $b_t^v = \boldsymbol{f}_4(\boldsymbol{a}_{t-1}, \boldsymbol{o}_t)$. Consequently, the belief given available information b_{t-1}, $\boldsymbol{a}_{t-1}$, $\boldsymbol{o}_t$, is $b_t = \{\boldsymbol{f}_1(b_{t-1}), \boldsymbol{f}_4(\boldsymbol{a}_{t-1}, \boldsymbol{o}_t)\}$. Therefore, $\boldsymbol{f}_1$ and $\boldsymbol{f}_4$ can be approximated using an RNN's hidden and input weights in a single step.

To investigate the advantages of separating the computation of prediction and update steps into two neural modules, we can consider a task with more complicated transition dynamics. For example, we previously developed a task variant where the joystick controls acceleration (59). The belief here comprises three components: position, velocity, and acceleration. Still, only the velocity component is observable (by optic flow), and computing it involves weighing $b_{t|t-1}^v$ and $\boldsymbol{o}_t$. Unlike our current task, $b_{t|t-1}^v$ here is not dependent

on $\boldsymbol{a}_{t-1}$, instead, it requires integrating the velocity and acceleration components from b_{t-1}. Therefore, combining the two-step EKF into a single step is more complicated, and a single RNN may find it harder to approximate the EKF computation in a single step. Future work may involve designing architectures with specialized modules for prediction and update in this task, and comparing their behaviors with agents without this modularization and the behaviors of humans (59, 60).

Fig. S1

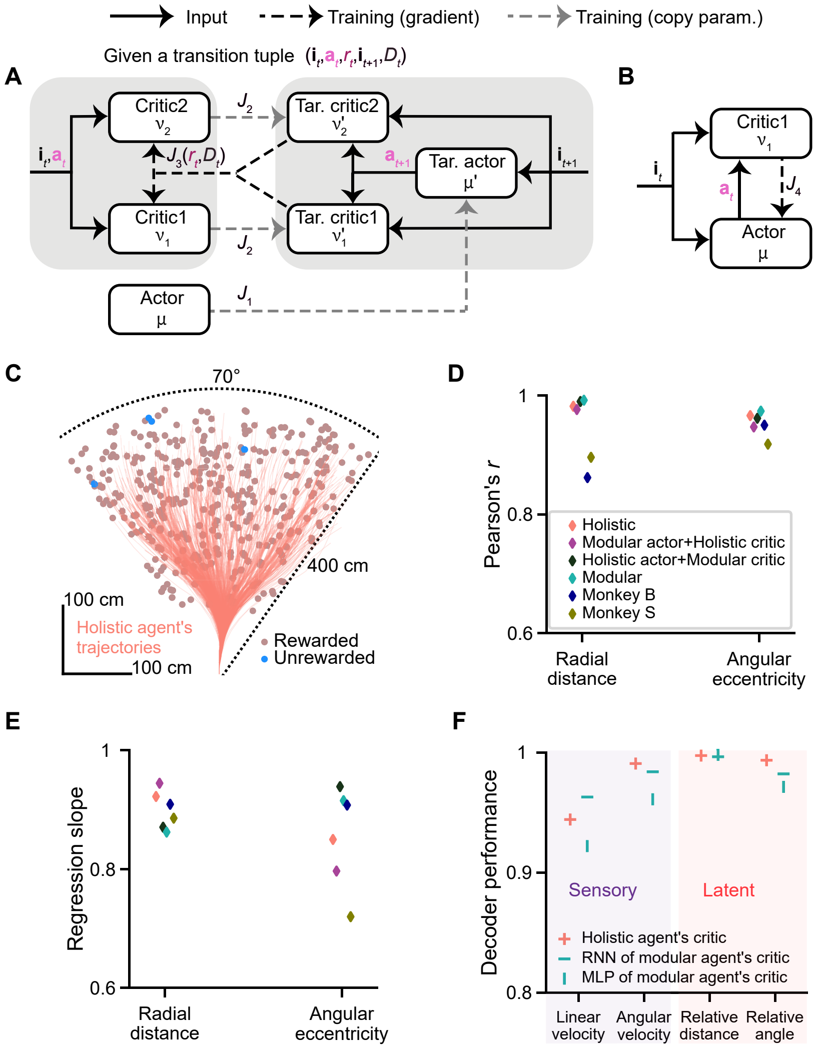

Figure S1: **RL agents with different neural architectures were trained in a partially observable navigation task.** (**A**) Critic training. The learning objective is to minimize the TD error, the difference between the value estimate of the current state-action and the TD target, i.e., reward plus the discounted value estimate of the next state-action. Given a transition tuple containing available information (state-related input $\boldsymbol{i}_t$, action $\boldsymbol{a}_t$, reward r_t, the next input $\boldsymbol{i}_{t+1}$, and trial completion flag D_t), theoretically, the next action $\boldsymbol{a}_{t+1}$ can be computed by the actor, and the next state-action value can be computed by the critic given $\boldsymbol{i}_{t+1}$ and $\boldsymbol{a}_{t+1}$. However, using these directly bootstrapped values in the TD target makes training very unstable. In practice, we implement more stable training using three trained networks and three target networks. Two critics and an actor have neural parameters ν_1, ν_2, and μ. Two target critics and a target actor have neural parameters ν_1', ν_2' and μ'. Target networks' parameters are copied from trained networks with an exponential moving average (learning objectives J_1 and J_2, Eq. 7). On the left side, each critic estimates the value of $\boldsymbol{a}_t$ in $\boldsymbol{i}_t$. On the right side, in $\boldsymbol{i}_{t+1}$, the target actor generates $\boldsymbol{a}_{t+1}$, and each target critic estimates value of $\boldsymbol{a}_{t+1}$ in $\boldsymbol{i}_{t+1}$. The TD target is constructed by these target networks, and ν_1, ν_2 are updated to minimize the TD error (J_3, Eq. 9). Critic training is off-policy, meaning $\boldsymbol{a}_t$ does not need to be the output of the up-to-date actor. See Materials and Methods for RL implementation details. (**B**) Actor training. Given $\boldsymbol{i}_t$, the actor's parameter μ is updated to generate $\boldsymbol{a}_t$ that maximizes the critic1's value (J_4, Eq. 10). (**C**) Similar to Fig. 1H, but showing an example holistic agent's trajectories. (**D**) Pearson correlation coefficient

for agents' and monkeys' stop locations versus target locations after training, for data shown in Fig. 1I. (**E**) Similar to (D), but showing regression slopes (>1/<1: over-/under-shooting). (**F**) Similar to Fig. 1L, but for modules in critics.

Fig. S2

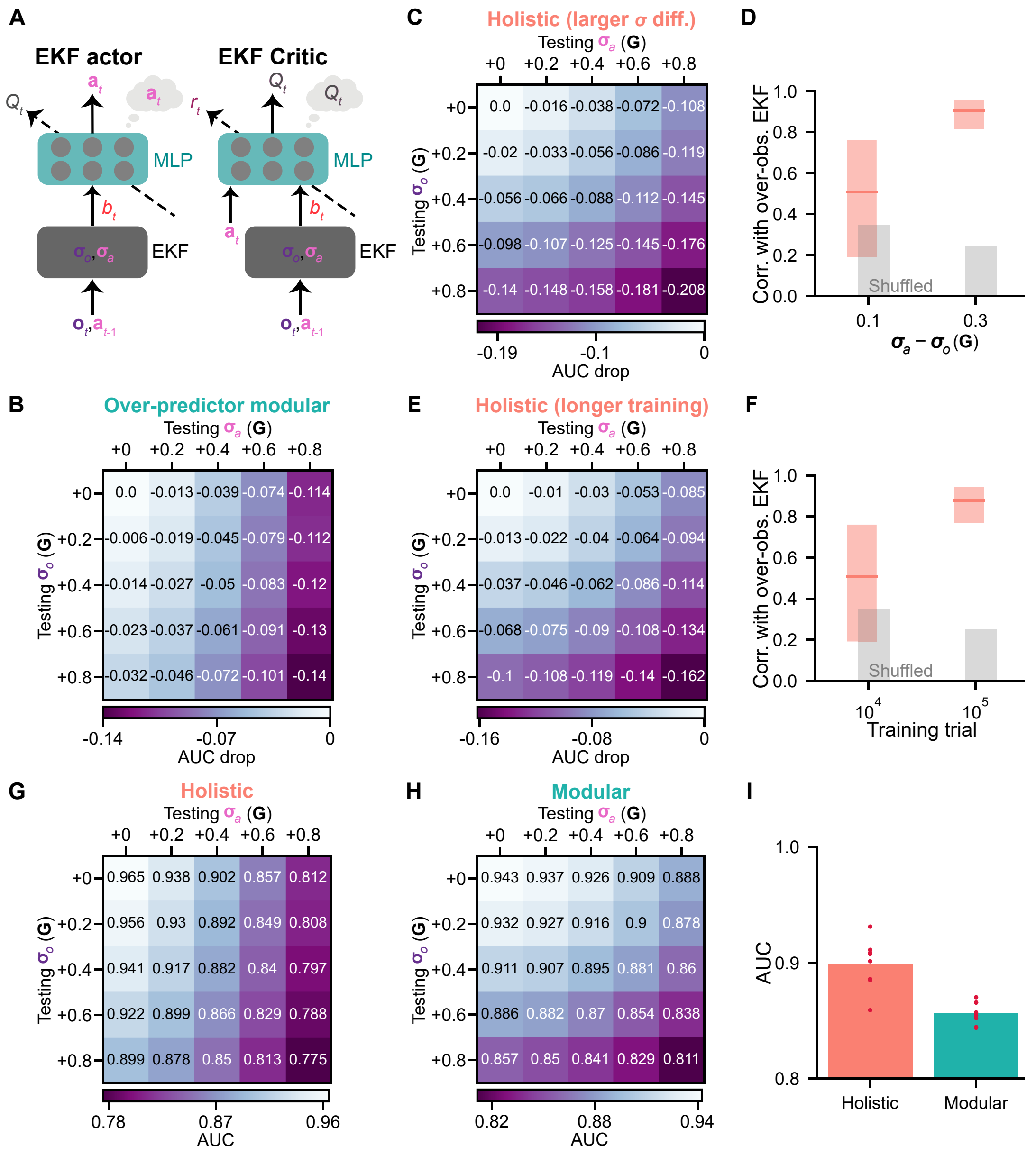

Figure S2: **The modular agent learned an EKF-like belief.** (**A**) Similar to Fig. 1, D and E, but showing the schematic of the EKF agent. An EKF module implements EKF computation to determine the belief b_t. Besides inputs o_t and a_{t-1}, this module also requires knowledge of uncertainties σ_o and σ_a. The computed belief is then fed into an MLP module to compute action in actor (*left*) or value in critic (*right*). Only the MLPs are trainable. (**B**) Similar to the bottom right panel in Fig. 2C, but this modular agent was trained with uncertainties $\boldsymbol{\sigma}_a = 0.1\boldsymbol{G}$ and $\boldsymbol{\sigma}_o = 0.2\boldsymbol{G}$, referred to as 'over-predictor modular'. (**C**) Similar to the top left panel in Fig. 2C, but this holistic agent was trained with uncertainties $\boldsymbol{\sigma}_a = 0.4\boldsymbol{G}$ and $\boldsymbol{\sigma}_o = 0.1\boldsymbol{G}$. (**D**) Correlation between the AUC drop of 'over-observer EKF' in Fig. 2B and that of the holistic agent trained with default uncertainties ($\boldsymbol{\sigma}_a - \boldsymbol{\sigma}_o = 0.1\boldsymbol{G}$; as shown in Fig. 2D) or with a larger uncertainty difference in (C) ($\boldsymbol{\sigma}_a - \boldsymbol{\sigma}_o = 0.3\boldsymbol{G}$). Error bars denote a 95% CI obtained through bootstrapping. Gray: correlation of shuffled data. (**E**) Similar to (C), but showing a holistic agent extensively trained for ten times the default duration used (default: 10^4, extensive: 10^5). (**F**) Similar to (D), but showing the extensively trained holistic

agent. (**G** and **H**) Similar to the holistic and modular agents in Fig. 2C, but showing the AUC instead of the AUC drop. (**I**) AUC of holistic and modular agents facing $+0.8\boldsymbol{G}$ in $\boldsymbol{\sigma}_o$ [bottom left corner in (G and H)]. Bars denote means across random seeds for each agent. Red dots denote data for individual seeds. $p = 0.001$ using the Mann–Whitney U test.

Fig. S3

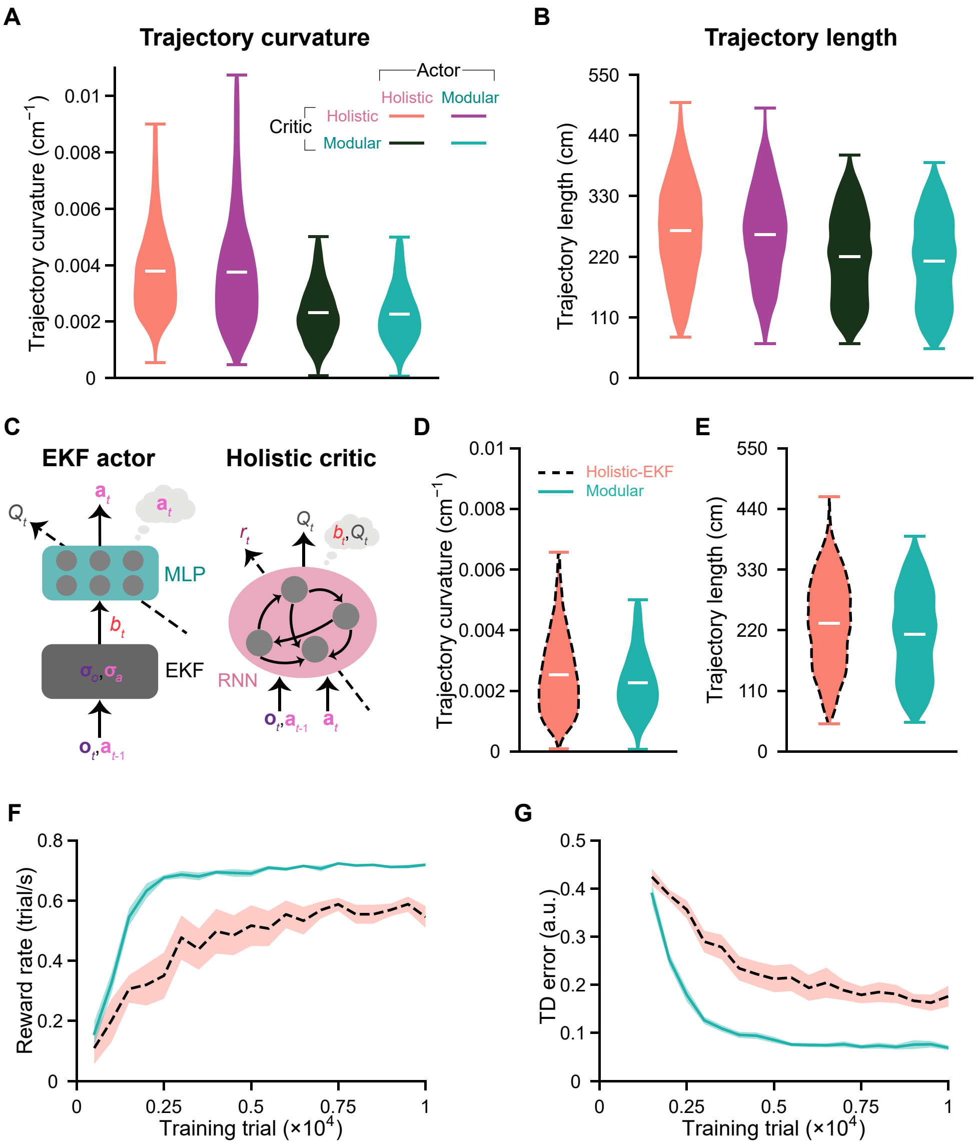

Figure S3: **Agents with modular critics exhibit superior efficiency and performance in learning.** (**A**) Distribution of trajectory curvature for agents navigating to the same set of 1000 targets. The curvatures were averaged across timesteps for each trajectory. (**B**) Similar to (A), but showing the length for each trajectory. (**C**) Similar to Fig. S2A, but showing the schematic of the holistic-EKF agent. This agent uses an EKF actor (Fig. S2A, left) and a holistic critic (Fig. 1E, left). (**D** and **E**) Similar to (A and B), but showing the holistic-EKF agent and the modular agent. (A, B, D, and E) Containing data from eight random seeds for each agent. White bars denote means across trials. (**F** and **G**) Similar to Fig. 3, C and D, but showing the holistic-EKF agent and the modular agent.

Fig. S4

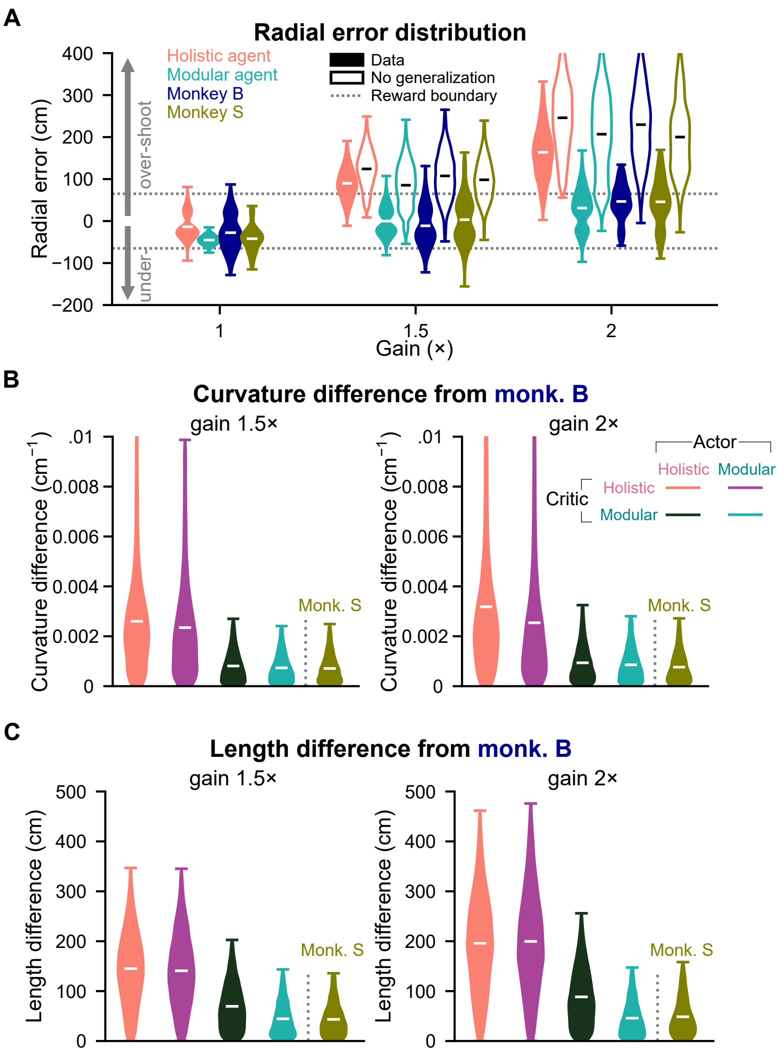

Figure S4: **Modular agent exhibits the best generalization performance in the gain task.** (**A**) Similar to Fig. 4, C and D, but showing detailed distributions of radial errors for agents and monkeys. (**B** and **C**) Similar to Fig. 3, A and B, but were conducted under two novel gain conditions. (A to C) Containing data from eight random seeds for each agent. White bars denote means across trials.

Fig. S5

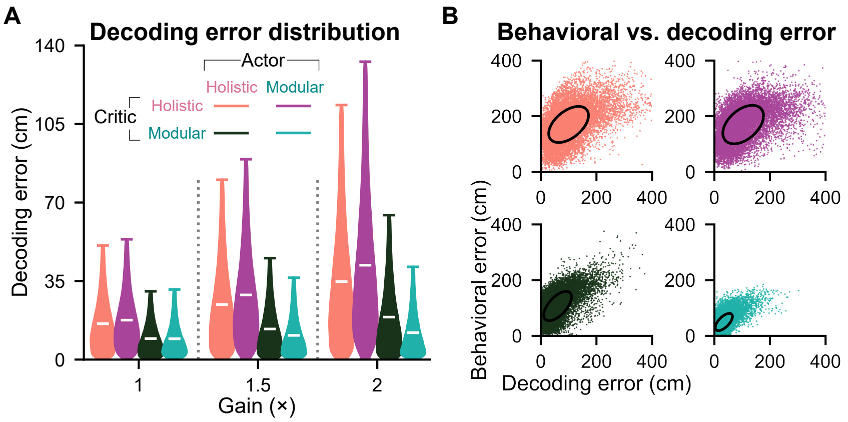

Figure S5: **Decoding error of agents' internal beliefs correlates with their behavioral performance in the gain task.** (**A**) Similar to Fig. 5D, but showing detailed distributions of decoding errors. White bars denote means across trials. (**B**) Behavioral error (absolute radial error between the target and the agent's stop locations) versus decoding error of stop locations (distance between decoded and true stop locations) for trials used in Fig. 5E. Confidence ellipses capture 1 SD. Pearson's r: Holistic, 0.50, Modular actor+Holistic critic, 0.45, Holistic actor+Modular critic, 0.61, Modular, 0.64, $p = 0$ for all agents. Regression slopes (without intercept): 1.28, 1.24, 1.31, 1.08. (A and B) Containing data from eight random seeds for each agent.

Fig. S6

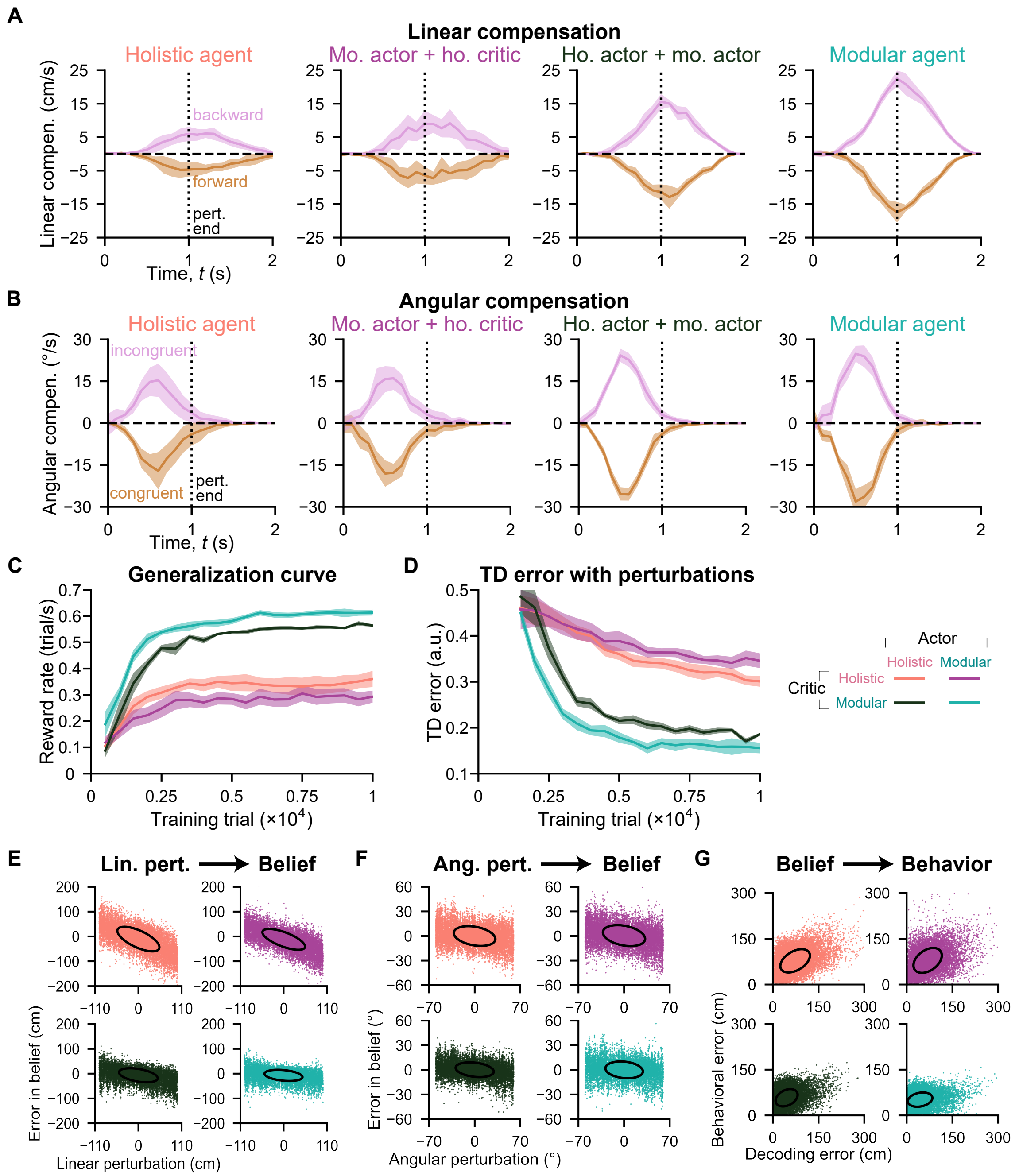

Figure S6: **Agents with modular critics exhibit better generalization performance and more accurate internal beliefs in the perturbation task.** (**A** and **B**) Agents' linear (A) and angular (B) compensatory actions (in units of velocities) in a 2 s time window, averaged across a test set comprising 1000 trials with consistent random target locations and perturbation parameters for each agent. Trials are aligned such that perturbations start at $t = 0$ s. The linear/angular compensatory actions are obtained by subtracting linear/angular actions in target-matched unperturbed trials from those in corresponding perturbation trials. Perturbations causing one to get closer to/further from targets are grouped as forward (+)/backward (−) for linear perturbations (A) and congruent (+)/incongruent (−) for angular perturbations (B). Vertical dotted lines denote the perturbation end time ($t = 1$ s). Horizontal dashed lines denote the null compensatory action.

Shaded regions denote ±1 SD across eight random seeds. (**C** and **D**) Similar to Fig. 3, C and D, but using a perturbation validation set. (**E** and **F**) Same as the top row of Fig. 6G, but showing all data points. Each dot denotes a trial. (**G**) Similar to Fig. S5B, but using trials in Fig. 6H. Pearson's r: 0.45, 0.40, 0.34, 0.23, $p = 0$. Regression slopes (without intercept): 0.85, 0.90, 0.89, 0.70.

Fig. S7

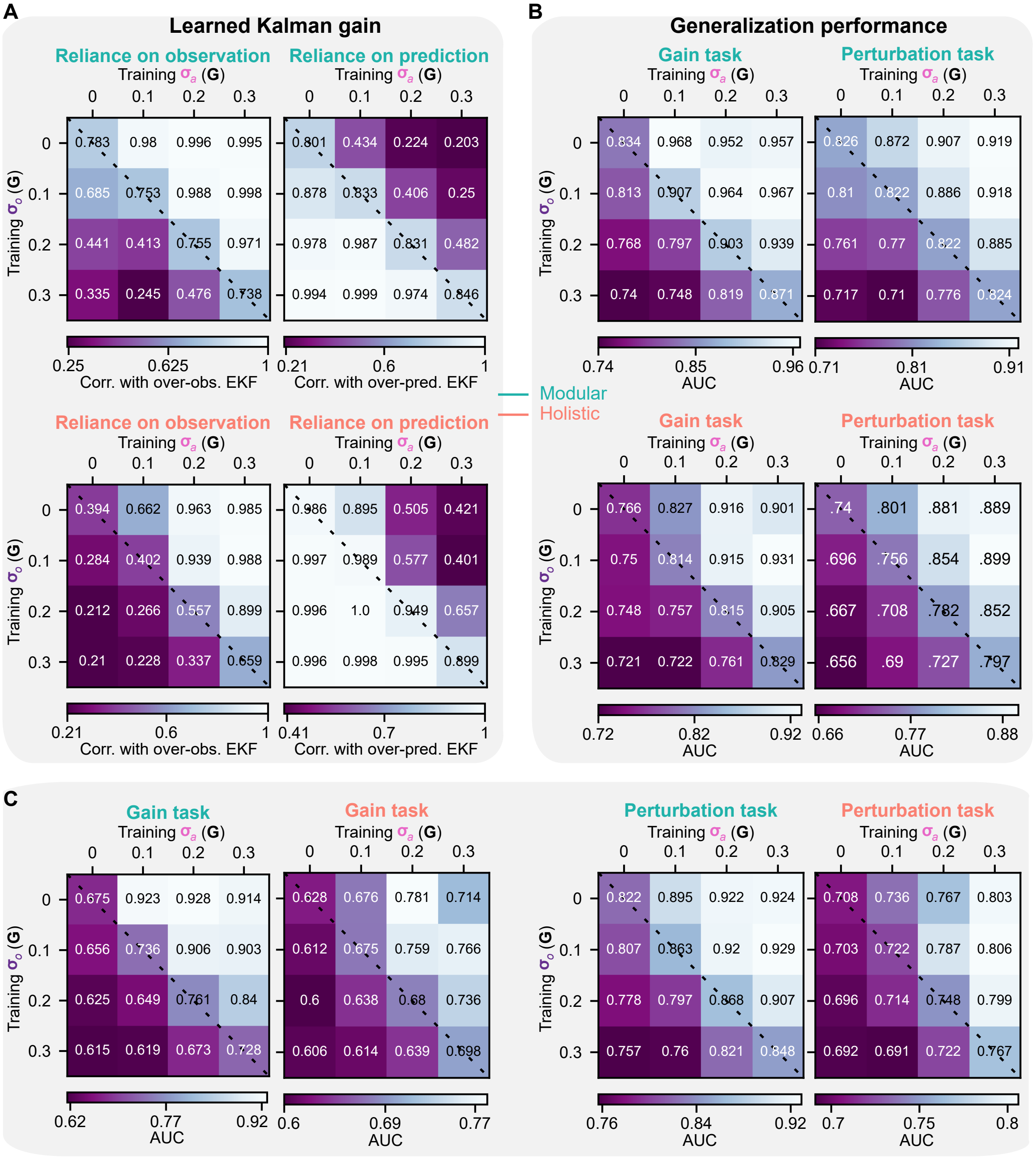

Figure S7: **Generalization in gain and perturbation tasks requires learning to rely more on observation.** (**A** and **B**) Similar to Fig. 7, A and B, but all agents were trained for ten times longer (10^5 trials). (**C**) Similar to (B), but in the gain task, gains were samples from $[3\times, 4\times]$; in the perturbation task, perturbation peak time was sampled from $[0.5, 1.5]$ s, and peaks of linear and angular perturbation velocities were sampled from $[-200, 800]$ cm/s and $[-180, 180]\,^{\circ}$/s, with smaller linear (within $[-100, 100]$ cm/s) and angular ($[-60, 60]\,^{\circ}$/s) perturbations excluded.

Fig. S8

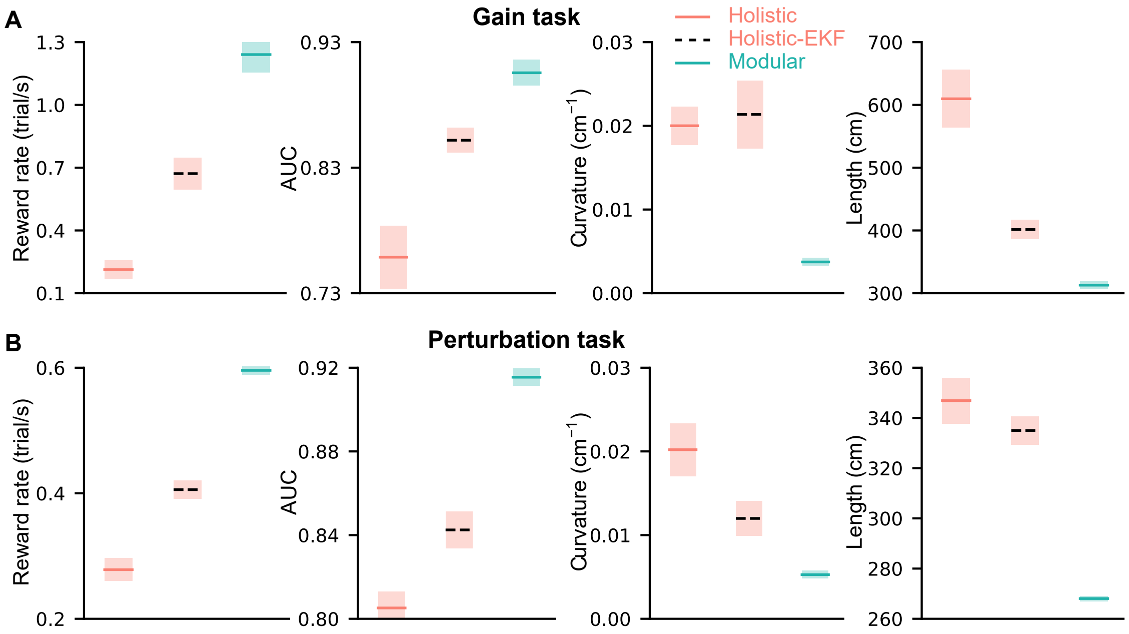

Figure S8: **An actor trained by the holistic critic improves generalization when using an EKF belief, but it remains inferior to the modular agent.** (**A**) Reward rate, AUC, trajectory curvature, and trajectory length for agents in the gain task, with gains sampled from $[3\times, 4\times]$. (**B**) Similar to (A), but agents were tested in the perturbation task, with perturbation peak time sampled from $[0.5, 1.5]$ s, and peaks of linear and angular perturbation velocities sampled from $[-400, 800]$ cm/s and $[-180, 180]\,^\circ$/s. (A and B) Lines denote means across eight random seeds, and shaded regions denote ± 1 SEM. For each seed of each agent in each task, 2000 trials were conducted.

Fig. S9

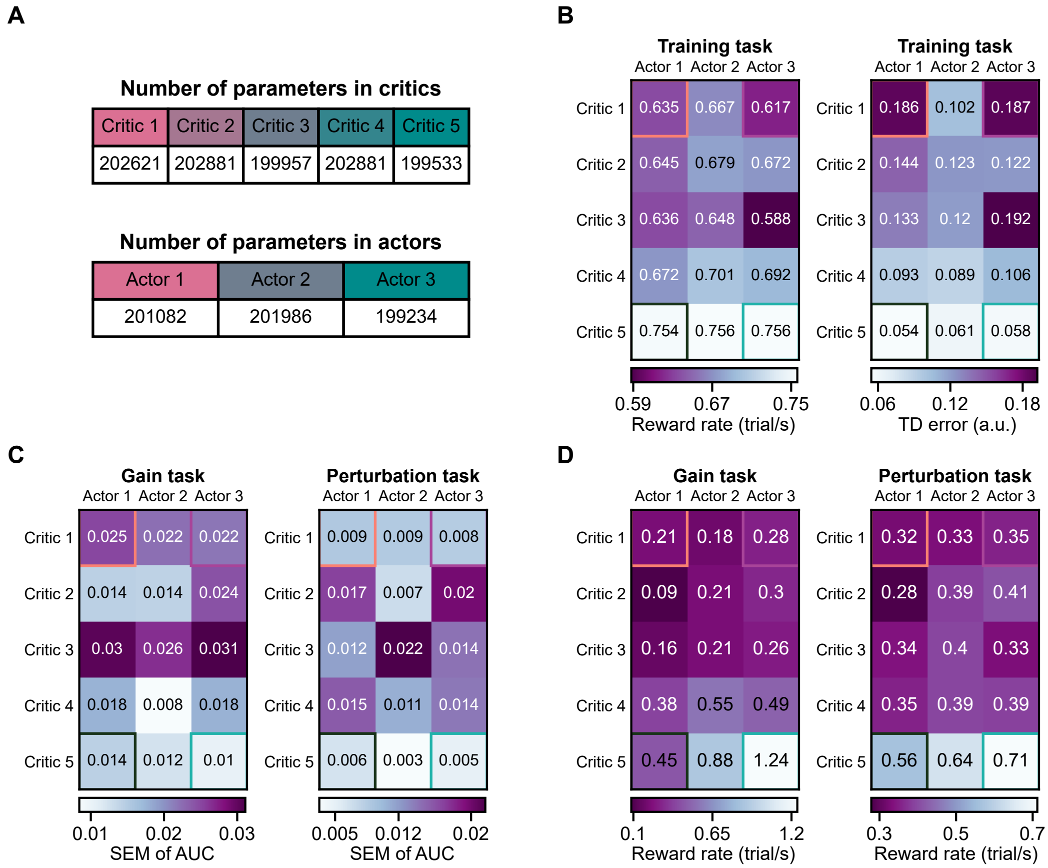

Figure S9: **Agents using less specialized modules exhibit less accurate internal beliefs, resulting in inferior performance compared to the modular agent.** (**A**) Total number of neural parameters for critics and actors in Fig. 8, A and B. (**B**) *Left*: Reward rate (rewarded trials/s) in 2000 training trials for agents with all combinations of actors and critics after training, averaged across eight random seeds for each agent. *Right*: TD error (averaged cross steps and trials) evaluated using the trials on the left. The four corners represent the four agents used in the previous analyses. Text in white/black denotes that the agent is worse/better than the average value of all agents. Pearson's r between left and right: -0.93, $p = 5.5 \cdot 10^{-7}$. (**C**) Similar to Fig. 8, C and D, top, but showing SEM of AUC across eight random seeds. (**D**) Similar to (B) left, but *left* uses gain trials from Fig. 8C, and *right* uses perturbation trials from Fig. 8D.

Table S1

Table S1: Hyperparameters for RL algorithms

discount factor γ	0.97
exploration σ_{exp}^2	$0.8 \to 0.5 \to 0.4$
buffer $\mathcal{B}$ size	1.6×10^6 transitions (EKF); 10^5 trajectories (RNN)
batch size M	256 transitions (EKF); 16 trajectories (RNN)
target networks update τ	0.005
ADAM learning rate	$3 \times 10^{-4} \to 5 \times 10^{-5}$
ADAM 1st, 2nd moment	0.9, 0.999
ADAM denominator term	1.5×10^{-4}
ADAM weight decay	0
critic update frequency c	every 4 environment interactions
actor update frequency	every 2 critic updates
target networks update frequency	every 2 critic updates
max trial duration	3.4 s
timestep Δt	0.1 s
max number of trials	10^4 (default training); 10^5 (extensive training)

Algorithm S1

Algorithm S1 EKF/RNN-TD3

```
Initialize network parameters μ, ν1, ν2, let target network parameters μ', ν1', ν2' ← μ, ν1, ν2
Initialize replay buffer B and optimizers for each network, let update timer t̃ = 0
for trial = 1 to max number of trials do
    Choose phase-specific σ_o, σ_exp, sample initial state s_0
    for t = 0 to max trial duration do
        Receive o_t, construct i_t with o_t, a_{t-1}
        Select a_t using Eq. 6 (EKF) / Eq. 11 (RNN)
        Receive r_t, o_{t+1}, D_t, construct i_{t+1} with o_{t+1}, a_t
        Store transition (i_t, a_t, r_t, i_{t+1}, D_t) and its mirror image in B (EKF only)
        if t̃ mod c = 0 then                                   ▷ Update critic for every c steps
            Sample transitions (EKF) / trajectories (RNN) from B
            Update ν1, ν2 by critic optimizers following Eqs. 8 and 9 (EKF) / Eqs. 12 and 13 (RNN)
        end if
        if t̃ mod 2c = 0 then                                  ▷ Update actor for every 2c steps
            Update μ by actor optimizer following Eq. 10 (EKF) / Eq. 14 (RNN)
            Update μ', ν1', ν2' using Eq. 7
        end if
        t̃ ← t̃ + 1
        if D_t = 1 then                                       ▷ Trial is done
            break
        end if
    end for
    Store trajectory (i_t, a_t, r_t, D_t)_{t=0,...,N-1} and its mirror image in B (RNN only)
end for
```

REFERENCES AND NOTES

1. T. E. Behrens, T. H. Muller, J. C. Whittington, S. Mark, A. B. Baram, K. L. Stachenfeld, Z. Kurth-Nelson, What is a cognitive map? Organizing knowledge for flexible behavior. *Neuron* **100**, 490–509 (2018).

2. D. Hume, *An Enquiry Concerning Human Understanding* (Routledge, 2016).

3. E. C. Tolman, Cognitive maps in rats and men. *Psychol. Rev.* **55**, 189–208 (1948).

4. H. F. Harlow, The formation of learning sets. *Psychol. Rev.* **56**, 51–65 (1949).

5. J. B. Tenenbaum, C. Kemp, T. L. Griffiths, N. D. Goodman, How to grow a mind: Statistics, structure, and abstraction. *Science* **331**, 1279–1285 (2011).

6. F. H. Sinz, X. Pitkow, J. Reimer, M. Bethge, A. S. Tolias, Engineering a less artificial intelligence. *Neuron* **103**, 967–979 (2019).

7. A. M. Zador, A critique of pure learning and what artificial neural networks can learn from animal brains. *Nat. Commun.* **10**, 3770 (2019).

8. A. Goyal, Y. Bengio, Inductive biases for deep learning of higher-level cognition. *Proc. R. Soc. A* **478**, 20210068 (2022).

9. S. Mittal, Y. Bengio, G. Lajoie, Is a modular architecture enough? *Adv. Neural. Inf. Process. Syst.* **35**, 28747–28760 (2022).

10. J. Peters, D. Janzing, B. Schölkopf, *Elements of Causal Inference: Foundations and Learning Algorithms* (The MIT Press, 2017).

11. Y. Bengio, T. Deleu, N. Rahaman, R. Ke, S. Lachapelle, O. Bilaniuk, A. Goyal, C. Pal, A meta-transfer objective for learning to disentangle causal mechanisms. arXiv:1901.10912 [cs.LG] (2019).

12. P. W. Battaglia, J. B. Hamrick, V. Bapst, A. Sanchez-Gonzalez, V. Zambaldi, M. Malinowski, A. Tacchetti, D. Raposo, A. Santoro, R. Faulkner, C. Gulcehre, F. Song, A.Ballard, J. Gilmer, G. Dahl, A. Vaswani, K. Allen, C. Nash, V. Langston, C. Dyer, N. Heess, D. Wierstra, P. Kohli, M. Botvinick, O. Vinyals, Y. Li, R. Pascanu, Relational inductive biases, deep learning, and graph networks. arXiv:1806.01261 [cs.LG] (2018).

13. D. H. Wolpert, W. G. Macready, No free lunch theorems for optimization. *IEEE Trans. Evol. Comput.* **1**, 67–82 (1997).

14. M. A. Bertolero, B. T. Yeo, M. D'Esposito, The modular and integrative functional architecture of the human brain. *Proc. Natl. Acad. Sci. U.S.A.* **112**, E6798–E6807 (2015).

15. S. Genon, A. Reid, R. Langner, K. Amunts, S. B. Eickhoff, How to characterize the function of a brain region. *Trends Cogn. Sci.* **22**, 350–364 (2018).

16. D. Meunier, R. Lambiotte, A. Fornito, K. Ersche, E. T. Bullmore, Hierarchical modularity in human brain functional networks. *Front. Neuroinform.* **3**, 37 (2009).

17. E. Bullmore, O. Sporns, Complex brain networks: Graph theoretical analysis of structural and functional systems. *Nat. Rev. Neurosci.* **10**, 186–198 (2009).

18. S. Grossberg, The complementary brain: Unifying brain dynamics and modularity. *Trends Cogn. Sci.* **4**, 233–246 (2000).

19. Y. LeCun, Y. Bengio, G. Hinton, Deep learning. *Nature* **521**, 436–444 (2015).

20. K. J. Lakshminarasimhan, E. Avila, E. Neyhart, G. C. DeAngelis, X. Pitkow, D. E. Angelaki, Tracking the mind’s eye: Primate gaze behavior during virtual visuomotor navigation reflects belief dynamics. *Neuron* **106**, 662–674.e5 (2020).

21. K. J. Lakshminarasimhan, E. Avila, X. Pitkow, D. E. Angelaki, Dynamical latent state computation in the male macaque posterior parietal cortex. *Nat. Commun.* **14**, 1832 (2023).

22. M. Watabe-Uchida, N. Eshel, N. Uchida, Neural circuitry of reward prediction error. *Annu. Rev. Neurosci.* **40**, 373–394 (2017).

23. R. S. Sutton, A. G. Barto, *Reinforcement Learning: An Introduction* (MIT press, 2018).

24. R. E. Kalman, A new approach to linear filtering and prediction problems. *J. Basic Eng.* **82**, 35–45 (1960).

25. J.-P. Noel, B. Caziot, S. Bruni, N. E. Fitzgerald, E. Avila, D. E. Angelaki, Supporting generalization in non-human primate behavior by tapping into structural knowledge: Examples from sensorimotor mappings, inference, and decision-making. *Prog. Neurobiol.* **201**, 101996 (2021).

26. P. Alefantis, K. J. Lakshminarasimhan, E. Avila, J.-P. Noel, X. Pitkow, D. E. Angelaki, Sensory evidence accumulation using optic flow in a naturalistic navigation task. *J. Neurosci.* **42**, 5451–5462 (2022).

27. J.-P. Noel, E. Balzani, E. Avila, K. J. Lakshminarasimhan, S. Bruni, P. Alefantis, C. Savin, D. E. Angelaki, Coding of latent variables in sensory, parietal, and frontal cortices during closed-loop virtual navigation. *eLife* **11**, e80280 (2022).

28. L. P. Kaelbling, M. L. Littman, A. R. Cassandra, Planning and acting in partially observable stochastic domains. *Artif. Intell.* **101**, 99–134 (1998).

29. S. Fujimoto, H. Hoof, D. Meger, Addressing function approximation error in actor-critic methods. *Proc. Mach. Learn. Res.* **80**, 1587–1596 (2018).

30. E. Balzani, K. Lakshminarasimhan, D. Angelaki, C. Savin, Efficient estimation of neural tuning during naturalistic behavior. *Adv. Neural. Inf. Process. Syst.* **33**, 12604–12614 (2020).

31. G. A. Einicke, L. B. White, Robust extended Kalman filtering. *IEEE Trans. Signal Process.* **47**, 2596–2599 (1999).

32. C. M. Bishop, Training with noise is equivalent to tikhonov regularization. *Neural Comput.* **7**, 108–116 (1995).

33. N. R. Ke, A. Didolkar, S. Mittal, A. Goyal, G. Lajoie, S. Bauer, D. Rezende, Y. Bengio, M. Mozer, C. Pal, Systematic evaluation of causal discovery in visual model based reinforcement learning. arXiv:2107.00848 [stat.ML] (2021).

34. V. Mnih, K. Kavukcuoglu, D. Silver, A. A. Rusu, J. Veness, M. G. Bellemare, A. Graves, M. Riedmiller, A. K. Fidjeland, G. Ostrovski, S. Petersen, C. Beattie, A. Sadik, I. Antonoglou, H. King, D. Kumaran, D. Wierstra, S. Legg, D. Hassabis, Human-level control through deep reinforcement learning. *Nature* **518**, 529–533 (2015).

35. K. L. Stachenfeld, M. M. Botvinick, S. J. Gershman, The hippocampus as a predictive map. *Nat. Neurosci.* **20**, 1643–1653 (2017).

36. A. Barreto, D. Borsa, J. Quan, T. Schaul, D. Silver, M. Hessel, D. Mankowitz, A. Zidek, R. Munos, Transfer in deep reinforcement learning using successor features and generalised policy improvement. *Proc. Mach. Learn. Res.* **80**, 501–510 (2018).

37. J. X. Wang, Z. Kurth-Nelson, D. Kumaran, D. Tirumala, H. Soyer, J. Z. Leibo, D. Hassabis, M. Botvinick, Prefrontal cortex as a meta-reinforcement learning system. *Nat. Neurosci.* **21**, 860–868 (2018).

38. M. Botvinick, S. Ritter, J. X. Wang, Z. Kurth-Nelson, C. Blundell, D. Hassabis, Reinforcement learning, fast and slow. *Trends Cogn. Sci.* **23**, 408–422 (2019).

39. J. A. Hennig, S. A. Romero Pinto, T. Yamaguchi, S. W. Linderman, N. Uchida, S. J. Gershman, Emergence of belief-like representations through reinforcement learning. *PLoS Comput. Biol.* **19**, e1011067 (2023).

40. J. Merel, D. Aldarondo, J. Marshall, Y. Tassa, G. Wayne, B. Ölveczky, Deep neuroethology of a virtual rodent. arXiv:1911.09451 [q-bio.NC] (2019).

41. V. Mikulik, G. Delétang, T. McGrath, T. Genewein, M. Martic, S. Legg, P. Ortega, Meta-trained agents implement bayes-optimal agents. *Adv. Neural. Inf. Process. Syst.* **33**, 18691–18703 (2020).

42. S. H. Singh, F. van Breugel, R. P. Rao, B. W. Brunton, Emergent behaviour and neural dynamics in artificial agents tracking odour plumes. *Nat. Mach. Intell.* **5**, 58–70 (2023).

43. T. Xu, O. Barak, Implementing inductive bias for different navigation tasks through diverse rnn attractors. arXiv:2002.02496 [q-bio.NC] (2020).

44. A. Zador, S. Escola, B. Richards, B. Ölveczky, Y. Bengio, K. Boahen, M. Botvinick, D. Chklovskii, A. Churchland, C. Clopath, J. D. Carlo, S. Ganguli, J. Hawkins, K. Körding, A. Koulakov, Y. L. Cun, T. Lillicrap, A. Marblestone, B. Olshausen, A. Pouget, C. Savin, T. Sejnowski, E. Simoncelli, S. Solla, D. Sussillo, A. S. Tolias, D. Tsao, Catalyzing next-generation artificial intelligence through neuroAI. *Nat. Commun.* **14**, 1597 (2023).

45. P. W. Glimcher, Understanding dopamine and reinforcement learning: The dopamine reward prediction error hypothesis. *Proc. Natl. Acad. Sci. U.S.A.* **108**, 15647–15654 (2011).

46. B. B. Doll, D. A. Simon, N. D. Daw, The ubiquity of model-based reinforcement learning. *Curr. Opin. Neurobiol.* **22**, 1075–1081 (2012).

47. D. Bennett, Y. Niv, A. J. Langdon, Value-free reinforcement learning: Policy optimization as a minimal model of operant behavior. *Curr. Opin. Behav. Sci.* **41**, 114–121 (2021).

48. W.-C. Jiang, S. Xu, J. T. Dudman, Hippocampal representations of foraging trajectories depend upon spatial context. *Nat. Neurosci.* **25**, 1693–1705 (2022).

49. C. Dan, B. K. Hulse, R. Kappagantula, V. Jayaraman, A. M. Hermundstad, A neural circuit architecture for rapid behavioral flexibility in goal-directed navigation. bioRxiv 456004 [Preprint] (2021). https://doi.org/10.1101/2021.08.18.456004.

50. M. Hadjiosif, J. W. Krakauer, A. M. Haith, Did we get sensorimotor adaptation wrong? Implicit adaptation as direct policy updating rather than forward-model-based learning. *J. Neurosci.* **41**, 2747–2761 (2021).

51. S. Thrun, A. Schwartz, Issues in using function approximation for reinforcement learning, in *Proceedings of the 1993 Connectionist Models Summer School* (Psychol. Dent. Press, 2014), pp. 255–263.

52. D. P. Kingma, J. Ba, Adam: A method for stochastic optimization. arXiv:1412.6980 [cs.LG] (2014).

53. S. Hochreiter, J. Schmidhuber, Long short-term memory. *Neural Comput.* **9**, 1735–1780 (1997).

54. M. Hausknecht, P. Stone, Deep recurrent q-learning for partially observable mdps. arXiv:1507.06527 [cs.LG] (2015).

55. B. Bakker, Reinforcement learning with long short-term memory. *Adv. Neural. Inf. Process. Syst.* **14**, 1475–1482 (2002).

56. B. Bakker, Reinforcement learning by backpropagation through an LSTM model/critic, in *2007 IEEE International Symposium on Approximate Dynamic Programming and Reinforcement Learning* (IEEE, 2007), pp. 127–134.

57. J. W. Tukey, *Exploratory Data Analysis* (1977).

58. H. Tang, R. Houthooft, D. Foote, A. Stooke, X. Chen, Y. Duan, J. Schulman, F. DeTurck, P. Abbeel, #Exploration: A study of count-based exploration for deep reinforcement learning. *Adv. Neural. Inf. Process. Syst.*, **30**, 2750–2759 (2017).

59. A. Stavropoulos, K. J. Lakshminarasimhan, J. Laurens, X. Pitkow, D. E. Angelaki, Influence of sensory modality and control dynamics on human path integration. *eLife* **11**, e63405 (2022).

60. A. Stavropoulos, K. J. Lakshminarasimhan, D. E. Angelaki, Belief embodiment through eye movements facilitates memory-guided navigation. bioRxiv 554107 [Preprint] (2023). https://doi.org/10.1101/2023.08.21.554107.
